# GPR133 (ADGRD1), an adhesion G-protein-coupled receptor, is necessary for glioblastoma growth

**DOI:** 10.1038/oncsis.2016.63

**Published:** 2016-10-24

**Authors:** N S Bayin, J D Frenster, J R Kane, J Rubenstein, A S Modrek, R Baitalmal, I Dolgalev, K Rudzenski, L Scarabottolo, D Crespi, L Redaelli, M Snuderl, J G Golfinos, W Doyle, D Pacione, E C Parker, A S Chi, A Heguy, D J MacNeil, N Shohdy, D Zagzag, D G Placantonakis

**Affiliations:** 1 Department of Neurosurgery, New York University School of Medicine, New York, NY, USA; 2 Kimmel Center for Stem Cell Biology, New York University School of Medicine, New York, NY, USA; 3 Department of Pathology, New York University School of Medicine, New York, NY, USA; 4 Genome Technology Center, New York University School of Medicine, New York, NY, USA; 5 Office for Therapeutic Alliances, New York University School of Medicine, New York, NY, USA; 6 Axxam SpA, Milan, Italy; 7 Brain Tumor Center, New York University School of Medicine, New York, NY, USA; 8 Perlmutter Cancer Center, New York University School of Medicine, New York, NY, USA; 9 Department of Neurology, New York University School of Medicine, New York, NY, USA

## Abstract

Glioblastoma (GBM) is a deadly primary brain malignancy with extensive intratumoral hypoxia. Hypoxic regions of GBM contain stem-like cells and are associated with tumor growth and angiogenesis. The molecular mechanisms that regulate tumor growth in hypoxic conditions are incompletely understood. Here, we use primary human tumor biospecimens and cultures to identify GPR133 (ADGRD1), an orphan member of the adhesion family of G-protein-coupled receptors, as a critical regulator of the response to hypoxia and tumor growth in GBM. GPR133 is selectively expressed in CD133+ GBM stem cells (GSCs) and within the hypoxic areas of PPN in human biospecimens. *GPR133* mRNA is transcriptionally upregulated by hypoxia in hypoxia-inducible factor 1α (Hif1α)-dependent manner. Genetic inhibition of GPR133 with short hairpin RNA reduces the prevalence of CD133+ GSCs, tumor cell proliferation and tumorsphere formation *in vitro*. Forskolin rescues the GPR133 knockdown phenotype, suggesting that GPR133 signaling is mediated by cAMP. Implantation of GBM cells with short hairpin RNA-mediated knockdown of GPR133 in the mouse brain markedly reduces tumor xenograft formation and increases host survival. Analysis of the TCGA data shows that *GPR133* expression levels are inversely correlated with patient survival. These findings indicate that GPR133 is an important mediator of the hypoxic response in GBM and has significant protumorigenic functions. We propose that GPR133 represents a novel molecular target in GBM and possibly other malignancies where hypoxia is fundamental to pathogenesis.

## Introduction

Glioblastoma (GBM) is a deadly brain malignancy with a poor prognosis.^1^ GBM growth, resistance to therapy and tumor recurrence are governed by a dynamic cellular hierarchy, in which GBM stem cells (GSCs) have a central role.^2, 3, 4, 5^ The molecular mechanisms that regulate GSC-mediated tumor growth are incompletely understood.

A cardinal histologic feature of GBM is intratumoral fluctuation in vascular density.^6^ Areas of microvascular proliferation are interspersed with hypoxic zones of pseudopalisading necrosis (PPN),^7^ a phenomenon suggesting that oxygen tension is variable within tumors. Previous literature suggested that GSCs, besides occupying vascular niches, may also reside within PPN.^8, 9, 10, 11, 12^ We, therefore, hypothesize that GSCs must entrain diverse molecular mechanisms to adapt to local oxygen tension and support tumor growth.

Recent literature has substantiated the concept that intratumoral hypoxia accelerates GBM growth. Hypoxia and acidity induce the stem cell phenotype.^13, 14, 15^ The hypoxia-induced transcription factors 1α and 2α (Hif1α and Hif2α) have been linked to tumor growth and invasiveness.^12, 16, 17, 18, 19^ Treatment-induced tumor hypoperfusion, as occurs in the majority of patients treated with the antiangiogenic agent cediranib, a vascular endothelial growth factor and its receptor inhibitor, correlates with worse survival compared with patients who respond with increased perfusion.^20^ Understanding the molecular mechanisms underlying hypoxia-driven tumor growth can provide novel molecular targets and improve outcomes following the antiangiogenic therapy.^21, 22^

Previous literature suggested that CD133-expressing tumor cell populations are enriched for stem-like cells with enhanced tumorigenic potential.^2, 23^ CD133+ GSCs are found not only in perivascular areas but also in the hypoxic areas of PPN.^10, 11, 12^ Therefore, profiling gene expression in CD133+ cells can reveal molecular signatures relevant to hypoxia-driven tumor growth.

Here, we report on the function of GPR133 (ADGRD1),^24, 25^ an orphan adhesion G-protein-coupled receptor (GPCR),^26^ which we found to be enriched in CD133-expressing GBM cells. Our data indicate an essential role for GPR133 in promoting GBM growth, especially in hypoxic conditions, and suggest that it may represent an appealing therapeutic target in GBM and possibly other malignancies where hypoxia is critical to pathogenesis.

## Results

### *GPR133* expression is upregulated in CD133+ GSCs

**Figure 1:**
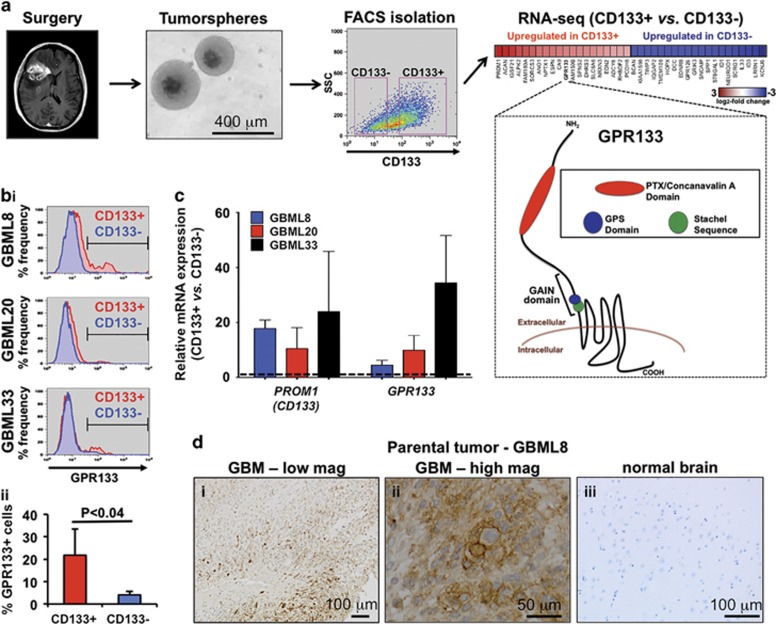
GPR133 is expressed in the CD133+ cell population of human GBM. (**a**) The experimental model consists of harvesting human GBM tissue during surgery and growing primary tumorsphere cultures. RNA-seq analysis of FACS-sorted CD133+ and CD133− cells from GBML8 in duplicates revealed 314 differentially expressed genes (fold-change cutoff: 1.5, false discovery rate (FDR)<0.05). GPR133 was among the top 20 genes overexpressed in CD133+ cells, as shown in the heatmap. GPR133's membrane topology and important domains within the extracellular N terminus are shown. (**bi**) Flow cytometry using a rabbit polyclocal antibody against GPR133 and a mouse monoclonal antibody against CD133 showed enrichment of GPR133 within the CD133+ cell population in three primary cultures. (**ii**) Cumulative statistics showing the percentage of GPR133+ cells within the CD133+ and CD133− populations in three cultures GBML8, GBML20 and GBML33 (*n*=3 experiments per culture, *P<*0.04, *t*-test; refer to Supplementary Figure 2 for individual statistics for each patient sample used). (**c**) Relative *CD133* and *GPR133* mRNA expression in FACS-isolated CD133+ and CD133− populations in three primary cultures (*n*=3 FACS experiments per culture). (**d**) Representative immunohistochemical analysis for *GPR133* expression in GBML8's parental tumor and normal brain tissue.

To identify novel genes that CD133+ GSCs require for tumorigenicity, we performed an RNA-sequencing (RNA-seq) comparison of FACS-sorted CD133+ and CD133− cells in duplicates from a primary human GBM culture, GBML8 (GEO accession number GSE85297) (Figure 1a).^27^ We identified 266 upregulated and 48 downregulated genes in CD133+ cells (Supplementary Tables 1a and b) (fold-change cutoff: 1.5; false discovery rate <0.05). *GPR133* was among the top 20 genes overexpressed in CD133+ cells (Figure 1a). GPR133 has a long N-terminal ectodomain, consisting of a signal peptide, a pentraxin/concanavalin A domain, and a GPCR-auroproteolysis-inducing domain, which includes a GPCR proteolysis site and the endogenous *Stachel* sequence agonist (Figure 1a).^28, 29, 30^ We hypothesized that GPR133 and its downstream effectors might represent a critical signaling pathway regulating tumorigenicity of CD133+ GSCs.

Enrichment of GPR133 in the CD133+ population was confirmed using flow cytometry (Figure 1bi and ii) and quantitative reverse transcriptase–PCR (qRT–PCR) (Figure 1c) in three primary cultures (GBML8, GBML20, GBML33; Supplementary Figure 1). Despite the variable percentage of CD133+ cells (35.44±19.52%), GPR133 surface expression was enriched in CD133+ cells when compared with CD133− cells by flow cytometry, using a commercially available rabbit polyclonal anti-GPR133 antibody (Figure 1bi and Supplementary Figures 2a and b). Enrichment was defined as the prevalence of GPR133+ cells in the CD133+ population compared with the prevalence of GPR133+ cells in the CD133− population. Within the CD133+ population, 21.77±11.72% of the cells were GPR133+, whereas in the CD133− population, only 4.06±1.48% of the cells expressed GPR133 (*n*=3 patient samples, *P<*0.04, *t*-test) (Figure 1bii and Supplementary Figures 2a and b). qRT–PCR revealed 16.30±9.22-fold upregulation of *GPR133* mRNA in CD133+ cells, along with 17.43±3.90-fold upregulation of *CD133* (*PROM1*) mRNA (Figure 1c). Analysis of human GBM biospecimens showed that 9/9 tumors expressed GPR133, using the rabbit polyclonal antibody. Representative images from GBML8's parental biospecimen are shown in Figures 1di–iii. The most intense immunostaining localized to the plasma membrane of tumor cells (Figure 1dii). *In silico* analysis using GTex Portal (http://www.gtexportal.org)^31^ showed that *GPR133* expression is very low in the human brain (Supplementary Figure 2c).^32^ Our immunohistochemistry also showed no staining in normal tissue from the cerebral hemisphere of patients (Figure 1diii), in agreement with a prior study that showed *GPR133* mRNA expression only in the pituitary gland and putamen within the brain^24^.

### *GPR133* mRNA is upregulated in hypoxia in Hif1α-dependent manner

**Figure 2:**
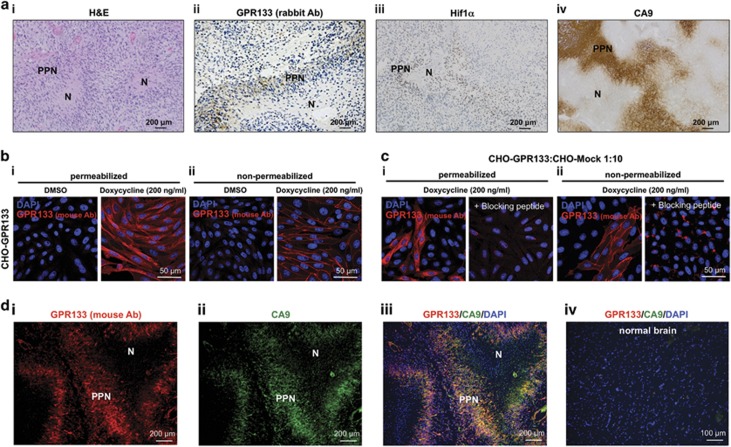
Expression of GPR133 overlaps with hypoxia markers in GBM biospecimens. (**ai**) Hematoxylin and eosin (H&E) of a human GBM showing the necrotic (N) and psesudopalisading necrosis (PPN) regions within the tumor. (**ii**–**iv**) Representative images of immunohistochemistry in human GBM biospecimens. A rabbit polyclonal anti-GPR133 antibody reveals selective expression of GPR133 in PPN (**ii**), along with hypoxic markers Hif1α (**iii**) and CA9 (**iv**). (**b**) Validation of mouse monoclonal GPR133 antibody using CHO cells inducibly overexpressing GPR133. (**i**) Permeabilized cells showed immunoreactivity at the cell membrane and intracellularly, likely reflecting trafficking of GPR133 along the secretory pathway. (**ii**) Immunoreactivity was confined to the cell membrane in non-permeabilized cells. In both (**i**) and (**ii**), GPR133 immunoreactivity was detected only after induction with doxycycline (200 ng/ml). (**c**) GPR133 immunoreactivity was abolished by the blocking peptide. (**d**, **i**–**iii**) Immunofluorescent analysis of GPR133 and CA9 in biospecimens. Costaining using a mouse monoclonal antibody against GPR133 in formalin-fixed paraffin-embedded (FFPE) biospecimens also confirms that *GPR133* expression highly overlaps with CA9 in (7/8 biospecimens, one biospecimen failed to show PPN). (**iv**) Normal brain shows neither CA9 nor GPR133 immunoreactivity (using the mouse monoclonal antibody). Ab, antibody.

Analyses of the nine human GBM biospecimens for *GPR133* expression with the rabbit polyclonal antibody revealed staining in areas of PPN, which are hypoxic and stain positive for the hypoxia markers Hif1α and carbonic anhydrase 9 (CA9) (Figures 2ai–iv).^33^ All samples also showed overlap between CD133 and *GPR133* expression (Supplementary Figure 3ai). Xenograft tumors generated from FACS-sorted CD133+ human GBM cells in NOD.SCID mice showed GPR133 immunoreactivity overlapping with hypoxyprobe (pimonidazole) staining, which identified hypoxic regions within poorly perfused regions of the tumor, as analyzed by intravenously injected Evans Blue^34^ (Supplementary Figures 3bi–iii). These findings suggest that *GPR133* expression is regulated by oxygen tension.

As we validated selective expression of GPR133 in hypoxic regions of GBM with additional immunostaining using a mouse monoclonal anti-GPR133 antibody, we developed against the extracellular pentraxin/concanavalin A domain (Supplementary Figure 4a). To validate this antibody, we immunostained CHO (Chinese hamster ovary) cell lines inducibly overexpressing GPR133 via a Tet-on promoter (CHO-GPR133). Overexpression of GPR133 in these cells was confirmed with qPCR (Supplementary Figure 4b). In CHO cells, our antibody stained cells only after induction with doxycycline (200 ng/ml). The staining of non-permeabilized CHO cells clearly identified GPR133 on the cell membrane, whereas in permeabilized cells GPR133 was also found intracellularly, reflecting trafficking of GPR133 along the secretory pathway (Figures 2bi and ii). Incubation of CHO cells with the immunizing peptide (blocking peptide, 8 μg/ml) prevented antibody binding (Figures 2ci and ii), indicating antibody specificity. CHO cells that were mock-transfected (CHO-Mock) showed no staining for GPR133 after doxycycline (Supplementary Figures 4ci–ii).

When we performed immunostaining of eight GBM biospecimens with the mouse monoclonal anti-GPR133 antibody and the anti-CA9 antibody, we observed that in 7/8 samples GPR133 colocalized with CA9 in hypoxic PPN regions (Figures 2di–iii and Supplementary Figure 3aii). One biospecimen failed to show any PPN regions; however, the CA9 and GPR133 staining still overlapped. Normal cerebral hemisphere tissue did not show any GPR133 immunoreactivity, as also seen with the rabbit polyclonal anti-GPR133 antibody (mouse antibody: Figure 2div; rabbit antibody: Figure 1diii). Collectively, these findings strongly suggest that *GPR133* expression is driven by hypoxia.

**Figure 3:**
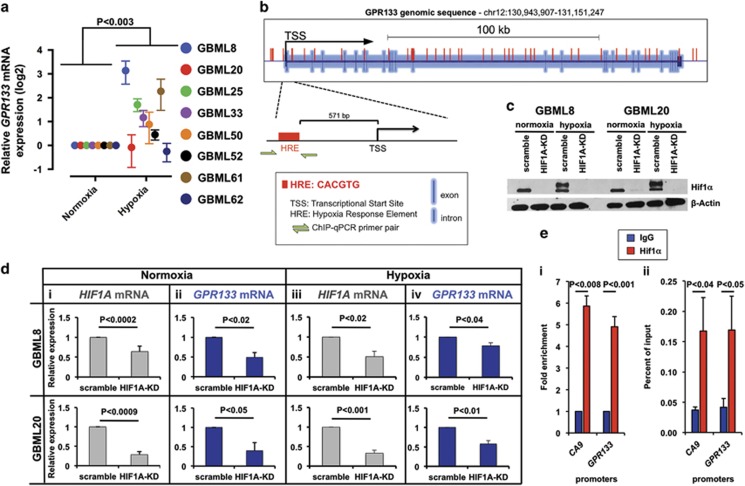
*GPR133* expression is upregulated by hypoxia. (**a**) Hypoxia upregulates *GPR133* mRNA in 6/8 primary cultures (*n*=3 measurements per culture; ANOVA F_(1,12)_=14.82; *P<*0.003). (**b**). Analysis of the GPR133 genomic locus reveals numerous HRE motifs, including immediately upstream of the TSS. The schematic also shows primers used for Hif1α ChIP-PCR experiment (green arrows). (**c**) Western blot shows effects of Hif1α knockdown (HIF1A-KD) on Hif1α protein levels in two primary cultures. β-Actin was used as a loading control. (**d**) Hif1α knockdown downregulates *HIF1A* and *GPR133* mRNA under normoxic (**i**, *HIF1A*: *n*=3 experiments per culture, *t*-test, *P<*0.002; and **ii**, *GPR133*: *n*=3 experiments per culture *t*-test, *P<*0.05) and hypoxic conditions (**iii**, *HIF1A*: *n*=3 experiments per culture, *t*-test, *P<*0.02; and **iv**, *GPR133*: *n*=3 experiments per culture, *t*-test, *P<*0.04) in two primary cultures: GBML8 (top row) and GBML20 (bottom row). (**e**) Fold enrichment (**i**) and percent of input (**ii**) representations of ChIP-PCR using Hifα antibody reveal that *GPR133*′s promoter region containing the HRE binds Hifα directly (**i**, *n*=3 primary cultures, two-tailed *t*-test, *P<*0.001; **ii**, *n*=3 primary cultures, one-tailed *t*-test, *P<*0.05). *CA9* promoter was used as a positive control (**i**, *n*=3 primary cultures, two-tailed *t*-test, *P<*0.008; **ii**, *n*=3 primary cultures, one-tailed *t*-test, *P<*0.04). Immunoglobulin G (IgG) alone was used as a negative control.

To test this hypothesis, we subjected GBM cultures to *in vitro* hypoxia (1% O_2_) for 24 h. We found significant upregulation of *GPR133* mRNA by hypoxia (3.01±1.06-fold), with 6/8 primary GBM cultures tested showing the effect (Figure 3a). *In silico* analysis of the genomic locus of *GPR133* revealed numerous hypoxia response elements (HREs; motif: CACGTG), including one 571 bp upstream of the transcriptional start site (TSS) (Figure 3b). To test whether Hif1α levels affect the expression level of *GPR133*, we knocked down Hif1α in two primary GBM samples using lentiviral short hairpin RNA (shRNA) (Supplementary Table 2c). This approach caused strong downregulation of Hif1α protein levels, as shown by western blot (Figure 3c), and *HIF1A* mRNA in normoxia (0.64±0.14 and 0.29±0.08 of control, in GBML8 and GBML20; Figure 3di) and hypoxia (0.49±0.12 and 0.40±0.21 of control, in GBML8 and GBML20; Figure 3diii). Upon Hif1α knockdown, we observed downregulation of *GPR133* mRNA to 0.49±0.12 and 0.40±0.21 of control in GBML8 and GBML20, in normoxia (Figure 3dii); and 0.79±0.07 and 0.58±0.09 of control in GBML8 and GBML20, in hypoxia (Figure 3div).

To test whether the *GPR133* promoter is directly bound to Hif1α, we performed chromatin immunoprecipitation-qPCR (ChIP-qPCR) assays in three primary cultures subjected to hypoxia (GBML8, GBML20 and GBML61), using Hif1α antibody and primers specific to the HRE 571 bp upstream of the TSS in the *GPR133* promoter (Figure 3b and Supplementary Table 2b). We observed significant enrichment of *GPR133* promoter upon immunoprecipitation with Hif1α antibody, compared with immunoglobulin G control (Figures 3ei and ii). The CA9 promoter region was used as a positive control. These results indicated that Hif1α transactivates *GPR133*.

### The long isoform of GPR133 is the predominant form in GBM

*In silico* analysis with UniProt (http://www.uniprot.org) revealed multiple putative GPR133 isoforms, varying in their N-terminal ectodomains. To investigate which isoform is relevant in GBM, we performed qRT–PCR on five primary GBM cultures using two different Taqman probes: one against the full N-terminal ectodomain (detecting only the long isoform) and one against the C-terminal cytoplasmic domain of *GPR133* (detecting all isoforms) (Supplementary Table 2a). We found no significant difference in mRNA levels amplified with either probe, suggesting that the full-length isoform is the predominant *GPR133* variant in GBM (Supplementary Figure 5a). This was supported by sashimi plots generated from RNA-seq data, which showed that the first and last exons of *GPR133* represented the most abundant reads (Supplementary Figure 5b).

### GPR133 knockdown depletes CD133+ GSCs, impairs *in vitro* tumorsphere formation and reduces cellular proliferation

**Figure 4:**
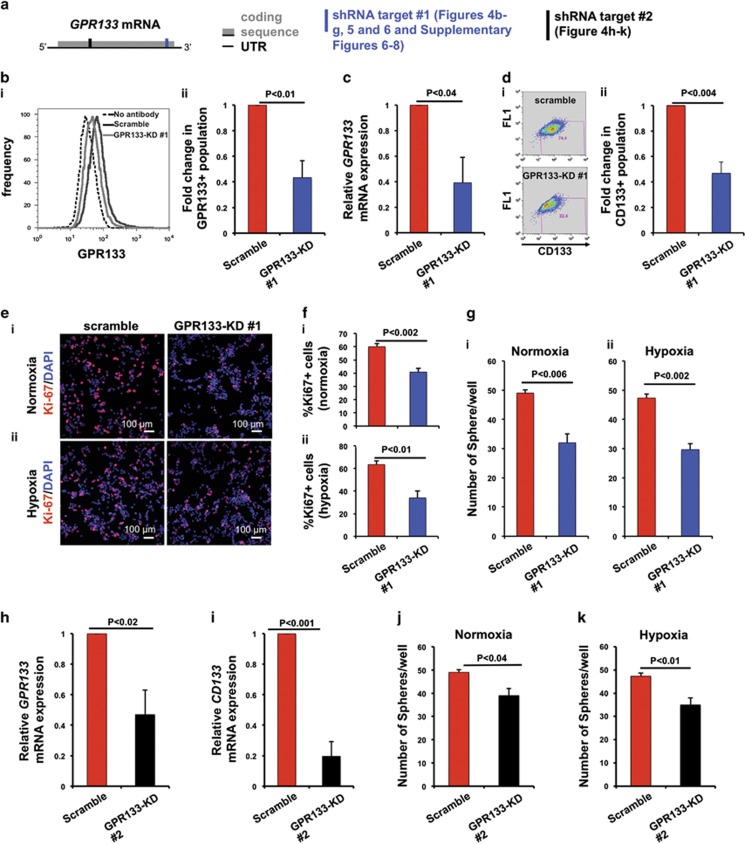
*In vitro* effects of GPR133 knockdown in GBML20. (**a**) Schematic of *GPR133* mRNA (full-length isoform) showing the recognition sites of the shRNA target sequences (target no. 1 in blue and target no. 2 in black) used. (**b**) GPR133 knockdown (GPR133-KD no. 1) reduces the number of GPR133+ cells, as shown by flow cytometry (*n*=3 experiments, *t*-test, *P<*0.01). (**c**) GPR133 knockdown (GPR133-KD no. 1) decreases *GPR133* mRNA levels (*n*=5, *t*-test, *P<*0.04). (**d**) Flow cytometry (**i**) indicates reduction in the abundance of CD133+ cells after GPR133 knockdown (GPR133-KD no. 1) (**ii**) (*n*=5 experiments, *t*-test, *P<*0.004). (**e** and **f**). Knockdown of GPR133 (GPR133-KD no. 1) reduces the percentage of Ki-67+ cells in both normoxia (**i**) (upper panel: *n*=3 experiments, *t*-test, *P<*0.002) and hypoxia (**ii**) (lower panel: *n*=3 experiments, *t*-test, *P<*0.01) (**g**). GPR133 knockdown impairs tumorsphere formation in normoxia (**i**) (*n*=3 experiments, *t*-test, *P<*0.006) and hypoxia (**ii**) (*n*=3 experiments, *t*-test, *P<*0.002). (**h** and **i**) GPR133 knockdown with the second shRNA construct (GPR133-KD no. 2) also reduces *GPR133* mRNA (**h**) (*n*=4 experiments, *t*-test, *P<*0.02) and *CD133* mRNA (**i**) (*n*=3 experiments, *t*-test, *P<*0.02) levels. (**j** and **k**) GPR133-KD no. 2 impairs tumorsphere formation in normoxia (**j**) (*n*=3 experiments, *t*-test, *P<*0.04) and hypoxia (**k**) (*n*=3 experiments, *t*-test, *P<*0.01) in GBML20.

The hypoxia-dependent regulation of *GPR133* expression suggests that signaling mediated by GPR133 could be critical for self-renewal of CD133+ GSCs, which reside in hypoxic microenvironments of GBM.^11^ To test this hypothesis, we used lentiviral shRNA-mediated knockdown of GPR133 in GBML20 and GBML8 (Figure 4 and Supplementary Figure 6, respectively). The knockdown constructs against GPR133 were designed to target the 3′ end of the coding portion (target no. 1), as well as the 5′ portion (target no. 2) of the *GPR133* mRNA (Figure 4a). We tested *GPR133* expression levels in cells lentivirally infected with the knockdown construct no. 1 (GPR133-KD no. 1), compared with a scrambled shRNA control. We observed reduced GPR133 expression, as analyzed by flow cytometry (GBML20: 0.43±0.13 of control (Figure 4b); GBML8: 0.59±0.06 of control (Supplementary Figure 6a). Knockdown was further confirmed with *GPR133* mRNA qRT–PCR (GBML20: 0.39±0.20 of control (Figure 4c); GBML8: 0.08±0.06 of control (Supplementary Figure 6b)). In line with the hypothesis that GPR133 is critical for self-renewal of CD133+ GSCs, we observed that GPR133-KD no. 1 led to a decrease in CD133+ GSCs (GBML20: 0.47±0.09 of control (Figure 4d); GBML8: 0.43±0.06 of control (Supplementary Figure 6c)).

We next tested the proliferative capacity of GBM cells. GPR133-KD no. 1 GBM cells had reduced proliferation measured by Ki-67 immunostaining under both normoxia (GBML20: 0.67±0.02 of control) and hypoxia (GBML20: 0.56±0.10 of control) (Figures 4e and f). These results were reproducible with GBML8 (0.71±0.08 of control in normoxia; 0.75±0.09 of control in hypoxia) (Supplementary Figures 6d and e). Phospho-histone H3 immunostaining corroborated these observations (Supplementary Figure 7a for GBML8 and Supplementary Figure 7b for GBML20).

We then performed *in vitro* tumorsphere formation assays to test the clonogenic ability of GSCs. When we seeded cells in low densities (4 cells per μl), we observed that GPR133-KD no. 1 reduced the number of spheres compared to scramble control in normoxia (GBML20: Figure 4gi; GBML8: Supplementary Figure 6fi) and hypoxia (GBML20: Figure 4gii; GBML8: Supplementary Figure 6fii). In addition, we performed limiting dilution sphere formation assay^35^ upon GPR133 knockdown (GBML8 and GBML20; Supplementary Figures 8a and b, respectively). The probability of sphere formation, which reflects the stem cell frequency, was significantly decreased upon GPR133 knockdown in both normoxia (Supplementary Figures 8ai and bi) and hypoxia (Supplementary Figures 8aii and bii). The size of spheres formed did not change upon GPR133-KD no. 1 in either GBML8 or GBML20 (Supplementary Figures 8ci and ii). These results suggest that GPR133 is required for sphere initiation by GSCs in both normoxia and hypoxia.

To rule out off-target effects, we repeated some key experiments using a different shRNA construct, which targets the 5′ coding region of the *GPR133* transcript encoding the full-length N-terminal ectodomain (target no. 2; Figure 4a). Using this second shRNA construct in GBML20, we observed downregulation in *GPR133* and *CD133* mRNA levels (*GPR133*: 0.47±0.16 of control; *CD133*: 0.19±0.12 of control) upon knockdown (Figures 4h and i). We also observed diminished tumorsphere formation in both normoxia (Figure 4j) and hypoxia (Figure 4k).

### GPR133 acts via upregulation of cAMP

**Figure 5:**
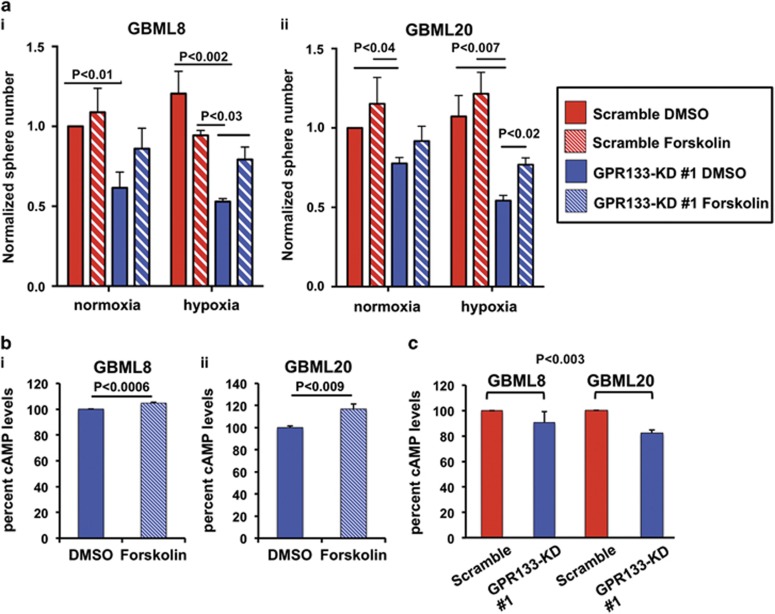
Forskolin rescues effects of GPR133 knockdown. (**a**) The GPR133-KD no. 1-induced impairment of tumorsphere formation in hypoxia is rescued by Forskolin treatment (10 μM) in GBML8 (**i**) (*n*=3 experiments, two-way ANOVA F_(3,6)=_7.726, *P<*0.02) and GBML20 (**ii**) (*n*=3 experiments, two-way ANOVA F_(3,6)_=9.655, *P<*0.01). Multiple comparisons were performed with Tukey's *post hoc* tests. (**b**) Ten micromolar Forskolin treatment increases cAMP levels in GBML8 (**i**) (*n*=3 experiments, *t*-test, *P<*0.0006) and GBML20 (**ii**) (*n*=3 experiments, *t*-test, *P<*0.009) under hypoxic conditions. (**c**) GPR133 knockdown decreases cAMP levels in GBML8 and GBML20 under normoxic conditions (*n*=3 experiments per condition, ANOVA F_(1,8)_=17.96, *P<*0.003).

GPR133 activates Gα_s_ and adenylyl cyclase *in vitro*, leading to cAMP elevation.^36^ To test whether GPR133 signaling in GBM is also mediated by cAMP, we used an activator of adenylyl cyclase, forskolin (10 μM), to rescue effects of GPR133-KD no. 1. Forskolin restored the impairment in tumorsphere formation under hypoxia in GBML8 (Figure 5ai) and GBML20 (Figure 5aii). Although not statistically significant, a similar rescue trend was observed under normoxic conditions. We used colorimetric assays to confirm that forskolin indeed raised intracellular cAMP levels in GPR133-KD no. 1 cells from GBML8 and GBML20 in hypoxia, thus validating the phenotypic rescue as a result of cAMP elevation (Figures 5bi and ii). Finally, we observed downregulation of cAMP after GPR133 knockdown in GBML8 (90.6±8.5% of control) and GBML20 (82.2±2.3% of control) (Figure 5c). These results argue that GPR133's signaling during the hypoxic response is mediated by cAMP, which is critical for tumorsphere formation.

### GPR133 knockdown impairs tumorigenicity

**Figure 6:**
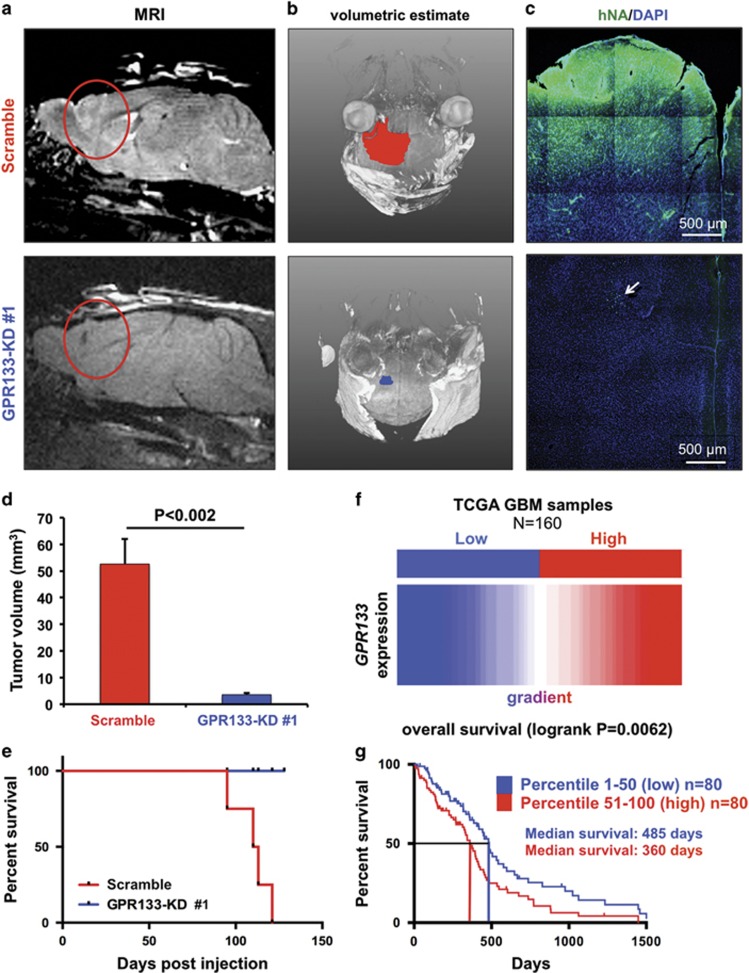
GPR133 knockdown prevents tumor formation and death *in vivo*. (**a** and **b**) Representative MRI images (**a**) and tumor volumetric estimates (**b**) show marked reduction in tumor xenograft size in mice implanted with GBM cells bearing GPR133 shRNA knockdown construct no. 1 (GPR133-KD no. 1) (GBML20, *n*=4 animals per group). (**c**) Histology of tumor xenografts. Tumor xenografts were identified by human nuclear antigen (hNA) immunoreactivity. The arrow indicates scattered tumor cells in the GPR133-KD condition, in the absence of formed tumor. (**d**) Cumulative statistics for tumor size obtained from MRI-based volumetric estimates (GBML20, *P<*0.002, *t*-test, *n*=4 animals per group). (**e**) Kaplan–Meier survival curves of the GPR133-KD no. 1 and control groups (log-rank Mantel–Cox test, *P*=0.0143). (**f**) The TCGA data from 160 patients with GBM were analyzed for *GPR133* expression. We studied outcomes in two cohorts GPR133 high (in red) and GPR133 low (in blue) based on ranked *GPR133* mRNA levels. (**g**) Kaplan–Meier curves of the two patient cohorts indicate an inverse relation between *GPR133* expression and survival (log-rank Mantel–Cox test, *P*=0.0062).

We tested *in vivo* tumorigenicity of GBM cells after GPR133 knockdown. GBML20 cells bearing either scramble or GPR133-KD no. 1 constructs were injected into the brains of NOD.SCID mice (5 × 10^5^ cells per animal, *n*=4 animals per group). Magnetic resonance imaging (MRI) and volumetric tumor analyses with the AMIRA software 1 month after injection revealed that control cells were able to form large tumors, as opposed to GPR133-KD no. 1 cells, which showed impaired tumor formation (Figures 6a and b). All control animals expired within 4 months after implantation, whereas GPR133-KD animals survived without any clinical deterioration. Histology at the time of death for scramble mice showed expansive and brain-infiltrating tumor formation in mice injected with scramble control GBM cells (Figure 6c, upper panel), whereas in animals injected with GPR133-KD no. 1 cells, we only found few scattered human cells, identified by human nuclear antigen staining at the site of the injection (Figure 6c, arrow in lower panel).

Cumulatively, quantitation of tumor volumes from MRI images showed that scramble controls formed significantly larger tumors, compared with GPR133-KD no. 1 cells (scramble: 52.6±9.40 mm^3^; GPR133-KD: 3.56±0.58 mm^3^; Figure 6d). Furthermore, GPR133-KD no. 1 led to a marked survival difference compared with animals injected with control GBM cells (Figure 6e). These results show that GPR133 is critical for tumorigenicity.

### *GPR133* expression correlates with poor prognosis in GBM

To understand the clinical relevance of our findings, we used the UCSC Cancer Genome Browser to analyze existing RNA-seq data from 160 GBM samples in the TCGA database.^37^ After ranking the samples according to expression levels of *GPR133*, we divided the samples into two groups (80 patients per group): GPR133 low (percentile 1–50, blue) and GPR133 high (percentile 51–100, red) (Figure 6f). We analyzed the survival of these two groups using Kaplan–Meier survival curves. In agreement with our mouse data, high *GPR133* expression correlates with poor prognosis and reduced survival (median survival 360 days vs 485 days in GPR133 high vs GPR133 low cohort) (Figure 6h). These data support the idea that GPR133 has protumorigenic function and suggest that its inhibition represents an attractive and novel therapeutic approach.

## Discussion

In GBM, hypoxia and its downstream cellular responses, such as increased stem-like phenotypes and angiogenesis, are associated with tumor progression.^38^ The idea that areas of PPN represent a hypoxic niche for CD133+ GSCs has been supported by previous literature.^11^ However, the key molecular events that regulate GSCs' response to hypoxia remain unclear.

Here, we show that GPR133 is enriched in PPN *in vivo* and CD133+ GSCs *in vitro*, regulated transcriptionally by oxygen tension and necessary for tumor growth. Although our analysis has shown significant overlap with CD133+ GSCs, CD133's reliability as a GSC maker has been questionable and the stem-like populations within GBM can also be identified using a variety of other markers.^5^ Further investigation is needed to understand the role of GPR133 in other stem-like populations within GBM and to test whether GPR133 itself is potentially a GSC marker.

The biology of GPR133 remains poorly understood, especially in the context of oncology, with our report being the first to link GPR133 with human cancer. Single-nucleotide polymorphisms in *GPR133* have been associated with adult height and the length of the RR interval in the cardiac electrical cycle.^39, 40, 41, 42^ Similar to other adhesion GPCRs, GPR133 features a large N-terminal extracellular domain, with a signal peptide and a pentraxin/concanavalin A domain. While the full mechanism of activation and the ligand are unknown, GPR133 activation is believed to involve autoproteolytic cleavage at a GPCR proteolysis site within the extracellular GPCR-auroproteolysis-inducing domain of the protein.^28, 30^ Ligand binding is thought to cause conformational changes in the extracellular domain, which reveal a tethered agonist (*Stachel* sequence), leading to receptor activation.^28^ *In vitro* assays revealed that GPR133 couples with Gα_s_, thereby activating adenylyl cyclase and leading to accumulation of cAMP.^36^ However, the relative contributions of intracellular signaling via G proteins vs cell-to-cell signaling via the N-terminal ectodomain have not been determined.

Our experiments show that hypoxia induces upregulation of *GPR133* mRNA via direct transcriptional activation by Hif1α. This regulatory mechanism is consistent with GPR133's localization in PPN regions, which are enriched for Hif1α and CA9.^33^ We observed this induction of *GPR133* mRNA by hypoxia in the majority of primary cultures we tested. The fact that some cultures do not show this phenomenon is consistent with the well-known concept of intertumoral heterogeneity and raises the question of whether the molecular components of the hypoxic response, including Hif1α, may differ across tumors.

We also found that reduced *GPR133* expression decreases the number of CD133+ GSCs, tumorsphere formation and cellular proliferation *in vitro*. The observation that GPR133 knockdown has effects in both normoxia and hypoxia *in vitro* likely reflects baseline expression levels of GPR133 in normoxic conditions. This is not surprising, as Hif1α protein levels in GBM cultures can be substantial, even in normoxia (Figure 3c).

Furthermore, our findings suggest that GPR133 signaling in GBM is mediated by elevations in cAMP. GPR133 knockdown reduces cAMP levels in GBM cells *in vitro.* Forskolin rescues the knockdown phenotype *in vitro*, in agreement with previous observations.^36^ The issue of how cAMP signaling regulates tumor growth in GBM has been relatively understudied and equivocal in prior literature.^43, 44, 45^ Further experiments will be needed to show how GPR133 signaling modulates gene expression, signaling and metabolism.

The effects of GPR133 knockdown on *in vivo* tumorigenesis were impressive. The knockdown condition abolished tumor initiation and prevented death in implanted mice. In agreement with our animal studies, analysis of the TCGA data show that increased expression in human GBM correlates with worse survival. These findings indicate a crucial role for GPR133 in GBM growth and suggest that it represents an appealing therapeutic target. Further experiments will be required to test whether genetic inhibition of GPR133 causes regression of tumors that have already formed.

In summary, GPR133 has an essential protumorigenic role in GBM, especially within hypoxic microenvironments. Given GPR133's important role in tumorigenesis in GBM, its cell surface localization and the general ‘druggability' of GPCRs, we propose that GPR133 is an enticing novel therapeutic target in GBM. We speculate that inhibition of GPR133 may be used in the future to target the hypoxic response that is triggered by, and likely accounts for the failure of, antiangiogenic therapy in GBM. Finally, our results may be applicable to other malignancies whose pathogenesis involves hypoxia.

## Materials and methods

### Patient biospecimens and primary tumor cultures

We used nine formalin-fixed paraffin-embedded patient biospecimens for immunohistochemistry or immunofluorescence, and nine fresh biospecimens to derive primary GBM tumorspheres, which were cultured in EGF/FGF2.^27, 46, 47^ We procured fresh tumor tissue from patients undergoing surgery for resection of GBM after informed consent (IRB no.12-01130). Molecular subtyping of parental tumors was performed with DNA methylation 450K arrays.^48^ Supplementary Figure 1 shows copy number variation profiles and key genomic/genetic changes in parental tumors.

### Flow cytometry

Cells were dissociated with Accutase (Innovative Cell Technologies, San Diego, CA, USA). CD133 staining was performed with APC-conjugated AC133 antibody (Miltenyi, Bergisch Gladbach, Germany; cat. no. 130-090-826; dilution 1:10). GPR133 analysis was performed using rabbit polyclonal anti-GPR133 antibody (LS Biosciences, Seattle, WA, USA), and goat anti-rabbit Alexa488-conjugated secondary antibody (Life Technologies, Carlsbad, CA, USA). The LSRII analyzer and FACSAria cell sorter (BD Biosciences, San Jose, CA, USA) were used for flow cytometry and FACS experiments.

### RNA-seq and bioinformatics

RNA was isolated from 30 000 FACS-isolated CD133+ and CD133− GBM cells using mirRNeasy Isolation Kit (Qiagen, Hilden, Germany). Data were represented as the average of two biological replicates from GBML8. Libraries were prepared using the Epicentre TotalScript RNA-Seq Kit (Illumina, San Diego, CA, USA), with oligo-(dT) as the primer for cDNA synthesis. The libraries were pooled equimolarly and run on a HiSeq 2500 sequencing system, as paired 50 nucleotide reads. Sequencing results were demultiplexed and converted to FASTQ format using Illumina Bcl2FastQ software (Illumina). Paired-end reads were aligned to the human genome (build hg19/GRCh37) using the splice-aware STAR aligner.^49^ PCR duplicates were removed using the Picard Toolkit (Broad Institute, Cambridge, MA, USA) (http://broadinstitute.github.io/picard). HTSeq package (Illumina) was used to generate counts for each gene based on how many aligned reads overlap its exons.^50^ These counts were then used to test for differential expression using negative binomial generalized linear models implemented by the DESeq2 R package.^51^ Isoform usage (sashimi plots and read coverage) via RNA-seq was visualized by plotting alignment data on the Integrative Genome Viewer (Broad Institute).

### Immunohistochemistry and immunofluorescence microscopy

All antibodies used, their dilutions and vendor information are listed in Supplementary Table 3. Chromogenic immunohistochemistry was performed on a Ventana Medical Systems Discovery XT instrument (Ventena Medical Systems, Tucson, AZ, USA) with online deparaffinization, using Ventana's reagents and detection kits (Ventana Medical Systems). Primary antibodies were detected by secondary antibodies conjugated to horse radish peroxidase (8 min). Complexes were visualized with 3,3′-diaminobenzidene and enhanced with copper sulfate.

For immunofluorescent analysis in formalin-fixed paraffin-embedded specimens, tissue was deparaffinized, blocked with 10% (w/v) bovine serum albumin (Sigma, St Louis, MO, USA) and 0.1% Triton X-100 (Sigma) in phosphate-buffered saline, and incubated with primary antibodies in blocking solution. CD133 signal was detected using biotinylated anti-mouse secondary antibody, followed by Alexa488-conjugated streptavidin combination (Life Technologies). Other primary antibodies were visualized with appropriate secondary antibodies conjugated to Alexa488 or Alexa555 (Life Technologies). Nuclei were stained with DAPI (4′,6-diamidino-2-phenylindole; Sigma).

For immunofluorescent microscopy analysis of tumor xenografts, animals were anesthetized with ketamine:xylazine (10 mg/kg; 100 mg/kg) and systemically perfused with first PBS and then 4% paraformaldehyde. Brain tissue was mounted in OCT (Tissue-Tek, Sakura Finetek, The Netherlands) and 30-μm-thick frozen sections were cut on a cryostat (Leica, Buffalo Grove, IL, USA). Sections were blocked and stained, as above. Pimonidazole staining was performed according to the manufacturer's protocol (Hypoxyprobe, Burlington, MA, USA).

The Eclipse E800 epifluorescent microscope (Nikon, Tokyo, Japan) and LSM700 confocal microscope (Zeiss, Oberkochen, Germany) were used for image acquisition. Image analyses were performed on ImageJ (NIH, Bethesda, MD, USA) and Adobe Photoshop (Adobe, San Jose, CA, USA).

### CHO cell lines inducibly overexpressing GPR133

The codon-optimized *GPR133* cDNA was subcloned into a Tet-on pcDNA3.1/TO/Neo vector (Axxam, Italy). This plasmid was stably transfected into CHO T-Rex cells (Axxam), which stably express rtTA (reverse tetracycline-controlled transactivator), using G418 (Sigma) selection. After clonal selection, one of the clones (CHO-GPR133 clone 11.6) that showed excellent inducibility was used for all experiments in this study. We used mock-transfected CHO T-Rex cells (CHO-Mock) as a negative control.

### Quantification of cellular proliferation in GBM primary cultures

Dissociated GBM cells were plated on Matrigel (Corning, Corning, NY, USA)-coated chamber slides, fixed, blocked/permeabilized and incubated with Ki-67 or phospho-histone H3 primary antibodies, followed by Alexa555-conjugated secondary antibody. Nuclei were counterstained with DAPI (Sigma). Quantification of the fraction of DAPI-positive nuclei that showed Ki-67 or phospho-histone H3 immunoreactivity was performed with the ImageJ software. Each condition was analyzed in biological triplicates. Four fields/condition were quantified in each experiment. A total of 1.3 × 10^4^ nuclei were counted.

### Validation of mouse monoclonal GPR133 antibody using CHO cells

We raised a mouse monoclonal antibody against GPR133′s N-terminal ectodomain (clone 8E3E8; Genscript), using this immunizing antigen: VNKGIYLKEEKGVTLLYYGRYNSSCISKPEQCGPEGVTFSFFWKTQGEQSRPIPSAYGGQVISNGFKVCSSGGR GSVELYTRDNSMTWEASFSPPGPYWTHVLFTWKSKEGLKVYVNGTLSTSDPSGKVSRDYGESNVNLVIGSEQDQAK.

The specificity of the antibody was tested using CHO-GPR133 and CHO-Mock cells. *GPR133* expression was induced with 200 ng/ml doxycycline (MP Biomedicals, Santa Ana, CA, USA) for 24 h. Immunofluorescence staining was performed as above. In some preparations, the blocking solution lacked Triton X-100, to ensure that CHO cells remained non-permeabilized.

### qRT–PCR expression analysis

RNA lysates and cDNA were prepared using TaqMan Gene Expression Cells-to-Ct Kit and the manufacturer's protocol (Ambion, Life Technologies). Fold changes in expression were calculated using the ΔΔCt method. The *HPRT1* and *GAPDH* genes were used for normalization. Supplementary Table 2a summarizes all Taqman assays used.

For *GPR133* qRT–PCR in CHO cells, RNA was isolated using the NucleoSpin RNA Kit (Macherey-Nagel, Duren, Germany). cDNA was generated with the High Capacity RNA-to-cDNA Kit (Applied Biotechnologies, Foster City, CA, USA). We used SYBR Green qPCR assays to quantitate *GPR133* cDNA (primers in Supplementary Figure 2b). Results were normalized to *18s* ribosomal RNA Taqman assays (Supplementary Table 2a) using the ΔCt method and were reported as relative expression units (REU = 2^−ΔCt^ × 10^7^).

### Stereotactic injections into mouse brain and MRI imaging

Male NOD.SCID mice (6–8 weeks) were stereotactically injected with 5 × 10^5^ GBM cells in the frontal lobe. Procedures were performed according to our IACUC-approved protocol (no. 120310-03), as described.^27^ Tumor formation was analyzed 1 month after injection with MRI.^27^ Stacked images were processed using ImageJ and Amira (FEI, Hillsboro, OR, USA) software.

### Pimonidazole and Evans Blue injections *in vivo*

Animals were injected with 60 mg/kg intraperitoneally pimonidazole (Hypoxyprobe) and 2% Evans Blue intravenously at 6 μl/g of body weight, 90 min and 5 min before killing, respectively. Brains were fixed and processed as described.^34^

### Hypoxia treatment

Cells were treated with hypoxic gas mixture (1% O_2_, 5%CO_2_, balanced with N_2_) for 24 h at 37 °C in a hypoxia chamber (Stem Cell Technologies, Vancouver, BC, Canada).

### ChIP-qPCR

EZ-Magna ChIP A/G Kit (Millipore, Billerica, MA, USA) was used for Hif1α ChIP-qPCR. GBM cells were subjected to hypoxia (1% O_2_) for 24 h, and crosslinked for 10 min with 1% paraformaldehyde. Pull-down was performed using a Hif1α ChIP-grade antibody (Abcam; cat. no. Ab2185). The HREs 571 bp upstream of the *GPR133* TSS and 3 bp upstream of the *CA9* TSS were quantified with qPCR (primers in Supplementary Table 2b). Data are depicted as fold enrichment relative to the immunoglobulin G control.

### Knockdown constructs

shRNA constructs against *GPR133* and *HIF1A*,^18^ as well as a scrambled shRNA sequence with no detectable targets in the human genome (Supplementary Table 2c), were cloned into the *Eco*RI and *Age*I sites of lentiviral vector pLKO.1_puro.

### Lentivirus production and transduction of GBM cultures

Lentiviruses were generated in Lenti-X 293 HEK (Clontech, Mountain View, CA, USA) producer cells, concentrated and titered with qPCR-based assays (ABM, Richmond, BC, Canada). Transduction of GBM cells was performed at multiplicity of infection of 5, as described.^27^ Three days after transduction, transduced cells were selected with 1 μg/ml puromycin (Life Technologies). Some experiments were performed 3 days after transduction without selection.

### Western blotting

Lysis buffer (150 mm NaCl, 50 mm Tris (pH 7.4), 1 mm EDTA, 0.1% Triton X-100, 10% glycerol) was supplemented with complete protease inhibitor cocktail (Roche, Basel, Switzerland). Nitrocellulose membranes were probed with anti-Hif1α (Bethyl Labs, Montgomery TX, USA) and anti-β-actin (Santa Cruz, Dallas, TX, USA) primary antibodies. Signal was detected with horse radish peroxidase-conjugated secondary antibodies (Life Technologies) and chemiluminescence (Thermo, Waltham, MA. USA).^27^

### cAMP measurements

cAMP levels were measured using cAMP-Glo assay (Promega, Madison, WI, USA). The manufacturer's protocol was followed.

### TCGA analysis

The UCSC Cancer Browser (https://genome-cancer.ucsc.edu) was used for the analysis of TCGA information on 160 GBM patients with RNA-seq data. Kaplan–Meier survival curves and statistical analysis were performed with Prism (Graphpad, La Jolla, CA, USA).

### Statistical analysis

Statistical comparisons included Student's *t*-test, one- and two-way analysis of variance (ANOVA) with *post hoc* Tukey's tests, and log-rank Mantel–Cox test for survival analysis. Statistical significance was set at *P<*0.05. Prism (GraphPad) and SPSS (IBM, Armonk, NY USA) were used for statistical analyses. Population statistics were represented as mean±s.e.
