# Supplementary material for: GPR133 (ADGRD1), an adhesion G-protein-coupled receptor, is necessary for glioblastoma growth
Source: Oncogenesis. 2016 Oct 24;5(10):e263–. doi: 10.1038/oncsis.2016.63 (PMC5117849; doi:10.1038/oncsis.2016.63)
Supplement: Supplementary Material [file oncsis201663x1.pdf]

Supplementary Figure and Table Legends

Supplementary Figure 1. DNA methylation-based classification of parental tumors. a. The parental tumors for 8/9 primary cultures used in this study were analyzed with 450k arrays for DNA methylation. Copy number variation (CNV) profiles are shown. **b.** Molecular subtype by DNA methylation and RNA-seq (where available), cytogenetic profile, important CNV information, IDH1 status and MGMT promoter methylation status are shown. NGS, next-generation sequencing.

Supplementary Figure 2. GPR133 expression information. a. Representative flow cytometry analysis of 3 primary GBM cultures for CD133 and GPR133. **b.** Summary statistics describing the abundance of GPR133+ and CD133+ cells in 3 primary cultures based on flow cytometric quantitation. **c.** Analysis of GTEx data on human tissue-specific expression of *GPR133*. Normal brain tissue expresses very low amounts of *GPR133*.

Supplementary Figure 3. Expression pattern of GPR133 in human GBM biospecimens and tumor xenografts. a. GPR133 immunoreactivity in hypoxic PPN regions of adjacent sections of an FFPE human GBM biospecimen, as detected with a

rabbit polyclonal (**i**) and a mouse monoclonal (**ii**) antibody. In (**i**), GPR133 expression overlaps with CD133 expression (arrowheads). In (**ii**), GPR133 and CA9 co-localize in PPN cells (arrowheads). **bi-iii.** GPR133 immunoreactivity co-localizes with pimonidazole in tumor xenografts in the mouse brain (n=3 animals). Evans Blue highlights the vasculature, which is relatively scant in the more hypoxic portion of the tumor staining positive for pimonidazole. The region of interest in (**ii**) is shown in high magnification in (**iii**), where several cells with overlapping GPR133 and pimonidazole staining are observed (arrowheads). N: necrosis; PPN: pseudopalisading necrosis.

Supplementary Figure 4. Characterization of a new mouse monoclonal anti-GPR133 antibody. a. The antibody recognizes the pentraxin/concanavalin A domain of GPR133's N-terminal ectodomain. **b.** CHO-GPR133 and mock-transfected cells (CHO-Mock) were subjected to qRT-PCR analysis for *GPR133* mRNA with and without doxycycline (1 μg/ml). **c.** In a quality control assay, CHO-Mock cells were immunostained with the anti-GPR133 mouse monoclonal antibody with and without doxycycline. Both permeabilized (**i**) and non-permeabilized (**ii**) preparations were used. We did not observe any GPR133 immunostaining.

Supplementary Figure 5. Quantitation of *GPR133* mRNA with two different Taqman assays and the distribution of reads obtained with RNA-seq reveal that full-length *GPR133* is the predominant isoform in GBM. a. The relative amounts of *GPR133* mRNA detected with two different Taqman assays did not differ, suggesting that the full-length *GPR133* splice variant is the dominant one in 5 primary GBM cultures (n=3 replicates/condition; t-test for each culture, P>0.05 throughout). **b.** Sashimi plots showing the distribution of reads obtained from RNA-seq comparison of FACS-isolated CD133+ and CD133- cells from GBML8 sample (average of 2 RNA-seq experiments shown). Reads were aligned with predicted *Ensembl* transcripts to show exon usage.

Supplementary Figure 6. *In vitro* effects of GPR133 knockdown in GBML8. The layout of this figure is identical to **Figure 4**, but describes data obtained with GBML8. **a. i.** Representative example of the effect of GPR133 knockdown (GPR133-KD#1) on the number of GPR133+ cells, as shown by flow cytometry. **ii**. Cumulative statistics on the reduction of GPR133+ cells by GPR133 knockdown (n=3 experiments, t-test, P<0.003). **b.** GPR133 knockdown decreases *GPR133* mRNA levels (n=3 experiments, t-test, P<0.0001). **c.** Flow cytometry indicates reduction in the abundance of CD133+ cells

after GPR133 knockdown. A representative example (**i**) and cumulative statistics (**ii**) (n=3 experiments, t-test, P<0.0005) are shown. **d,e.** Knockdown of GPR133 reduces the percentage of Ki67+ cells in both normoxia (n=3 experiments, t-test, P<0.02) and hypoxia (n=3 experiments, t-test, P<0.05). **e.** GPR133 knockdown impairs tumorsphere formation in (**i**) normoxia (n=3 experiments, t-test, P<0.002) and (**ii**) hypoxia (n=3 experiments, t-test, P<0.002).

Supplementary Figure 7. *In vitro* effects of GPR133 knockdown on cell proliferation. We tested the effects of GPR133 knockdown (GPR133-KD#1) on cell proliferation using immunostaining for phospho-H3 (pH3) in GBML8 (**a**) and GBML20 (**b**) cells *in vitro*. Representative images are shown in (**ai**) and (**bi**). Summary statistics in (**aii**) and (**bii**) indicate that GPR133 knockdown produces a significant reduction in pH3 staining, indicating reduced tumor cell proliferation (GBML8: n=3 experiments, t-test, P<0.001; GBML20: n=3 experiments, t-test, P<0.0001).

Supplementary Figure 8. Effects of GPR133 knockdown on limiting dilution assays and sphere size in GBML8 and GBML20. a,b. Dissociated GBML8 (**a**) and GBML20 (**b**) cells bearing either scrambled or GPR133-KD#1 constructs were plated in

limiting dilutions (1,10,100 and 1000 cells/well) and allowed to form spheres in either (**i**) normoxia or (**ii**) hypoxia. GPR133-KD in all conditions led to a significant reduction in the probability of sphere formation, indicating a reduction in stem cell frequency ($P<0.0001$). Along these lines, the number of spheres formed/well was significantly lower in (**i**) normoxia (GBML8: ANOVA $F_{(1,12)}=47.68$, $P<0.0001$; and GBML20: ANOVA $F_{(1,12)}=84.55$, $P<0.0001$) and (**ii**) hypoxia (GBML8: ANOVA $F_{(1,12)}=40.59$, $P<0.0001$; and GBML20: ANOVA $F_{(1,12)}=44.57$, $P<0.0001$). P values for *post hoc* multiple comparisons by Tukey's test are shown in the figure (n=5 wells/condition). **c**. Analysis of the size of the spheres formed using (**i**) GBML8 and (**ii**)**.** GBML20 cells did not reveal any significant difference.

Supplementary Table 1. a. Genes upregulated in CD133+ vs. CD133- GBM cells. **b.** Genes downregulated in CD133+ vs. CD133- GBM cells.

Supplementary Table 2. a. Taqman Assays, **b.** PCR primers and **c.** shRNA constructs used in the study.

Supplementary Table 3. Antibodies used for immunofluorescence staining.

Supplementary Figure 1

a

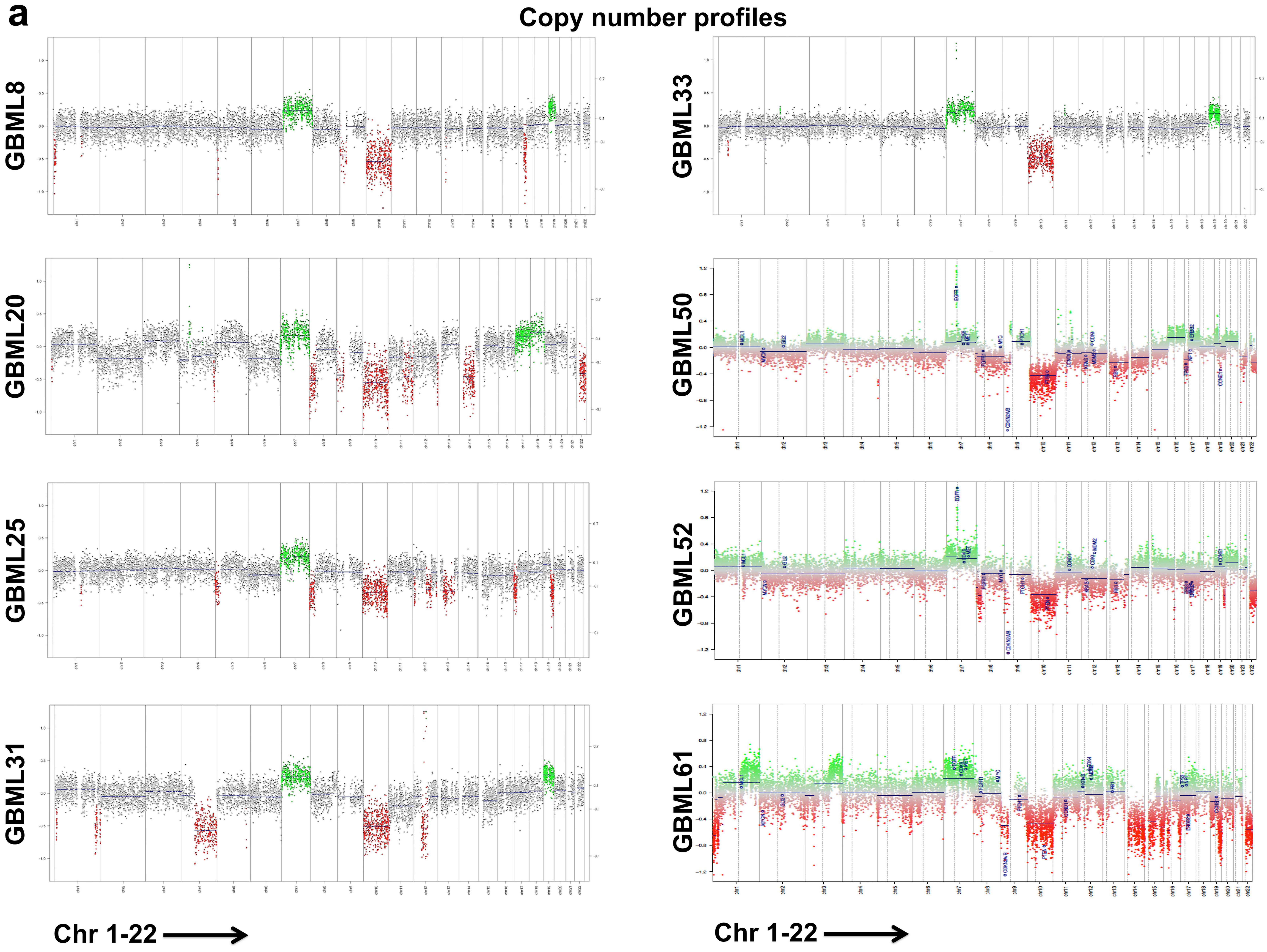

b

Patient sample	Score/subtype by DNA Methylation array	Score/subtype by RNA-seq	Cytogenetics	Other implied changes from CNV or NGS analysis	IDH1 status	MGMT methylation
**GBML8**	Mesenchymal	Mesenchymal	7+, 9p-, 10-, 17q-, 19p +	CDKN2A loss, PTEN loss, Nf1 loss	IDH wild-type	Methylated
**GBML20**	GBM RTK1 (proneural)	Proneural	7+, 8p-, 10-, 11q-, 14-, 17+	PDGFRA amp, PTEN loss	IDH wild-type	Not methylated
**GBML25**	GBM Classic/ Mesenchymal & GBM RTK1	Not available	4q-, 7+, 10-, 12q-, 12q+, 19+	PTEN loss, MDM2 amp	IDH wild-type	Not methylated
**GBML31**	GBM Classic	Not available	7+, 10-, 19+	EGFR amp, PTEN loss	IDH wild-type	Methylated
**GBML33**	GBM RTK1 (proneural)	Not available	5p-, 7+, 8p-, 10-, 12q-, 13q-, 17p-, 19q-	TERT loss, PTEN loss, Rb1 loss, TP53 loss	IDH wild-type	Not methylated
**GBML50**	GBM RTK1 (proneural)	Not available	10-	EGFR amp, CDKN2A loss	IDH wild-type	Not methylated
**GBML52**	GBM Classic/ Mesenchymal	Classic	7+, 8p-, 10-, 19q-, 22-	EGFR amp, CDKN2A loss, TP53 mutation, PTEN mutation	IDH wild-type	Not methylated
**GBML61**	GBM RTK1 (proneural)	Not available	1p-, 3q+, 7+, 9p-, 10-, 14-, 15q-, 19q-, 22-	CDKN2A loss, PTEN loss	IDH wild-type	Not methylated
**GBML62**	Not available	Not available	Not available	TP53 mutation, Rb1 mutation	IDH wild-type	Not methylated

Supplementary Figure 2

a

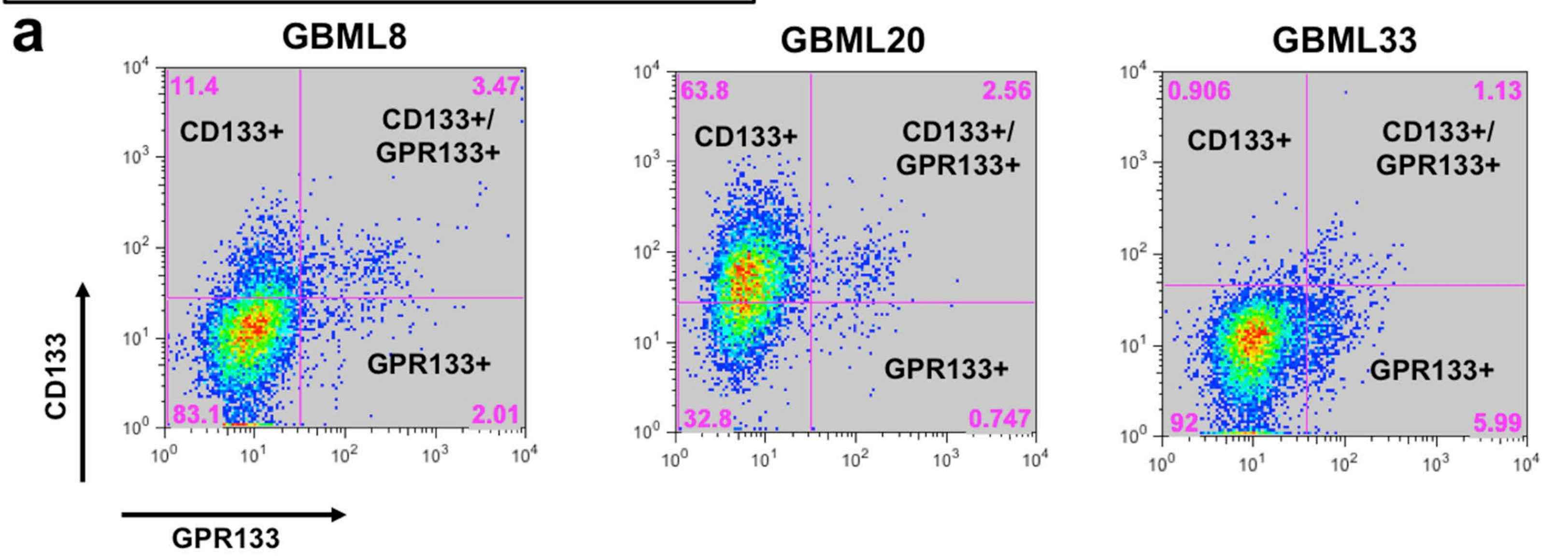

b

	% CD133+ cells	% GPR133+ cells	% GPR133+ cells in CD133+ population	% GPR133+ cells in CD133- population
GBML8	31.29 ± 16.00	2.95 ± 1.27	16.05 ± 4.78	1.79 ± 0.29
GBML20	71.13 ± 6.22	4.62 ± 0.86	4.92 ± 1.33	3.56 ± 1.09
GBML33	3.90 ± 2.51	9.39 ± 1.56	44.32 ± 13.32	6.84 ± 2.13
**Cumulative (n=3 samples)**	**35.44 ± 19.52**	**5.65 ± 1.92**	**21.77 ± 11.72**	**4.06 ± 1.48**

c

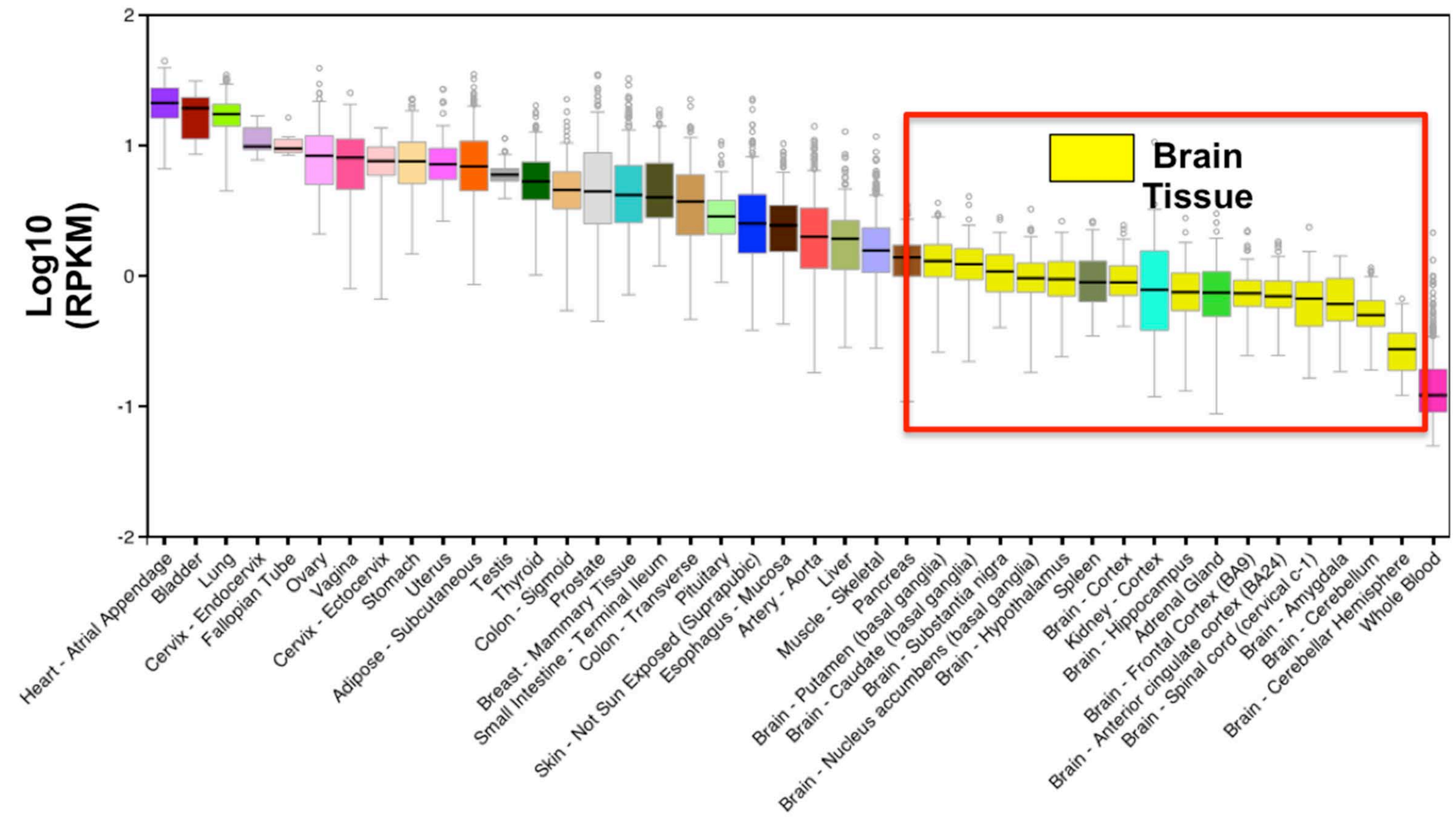

Supplementary Figure 3

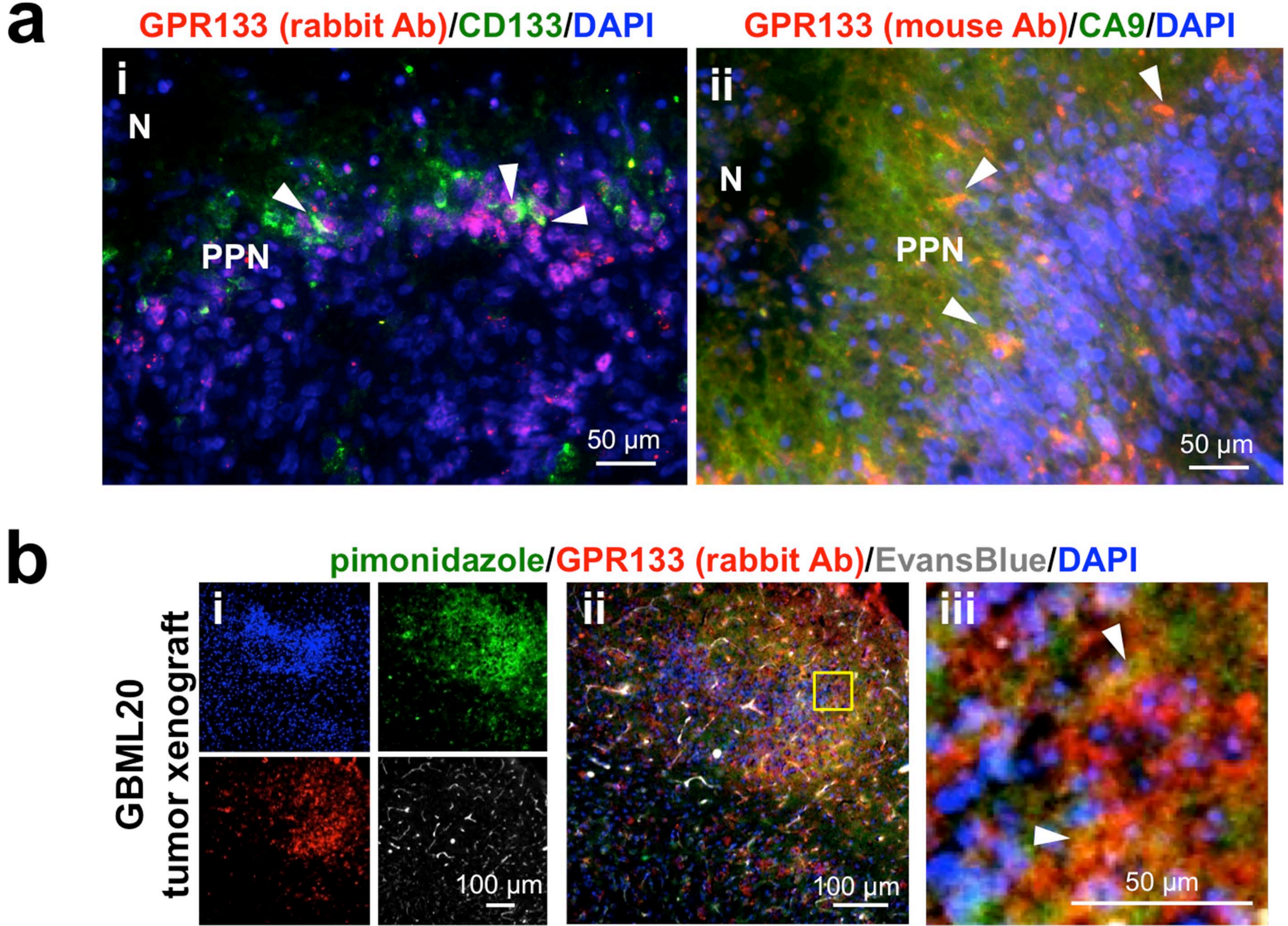

Supplementary Figure 4

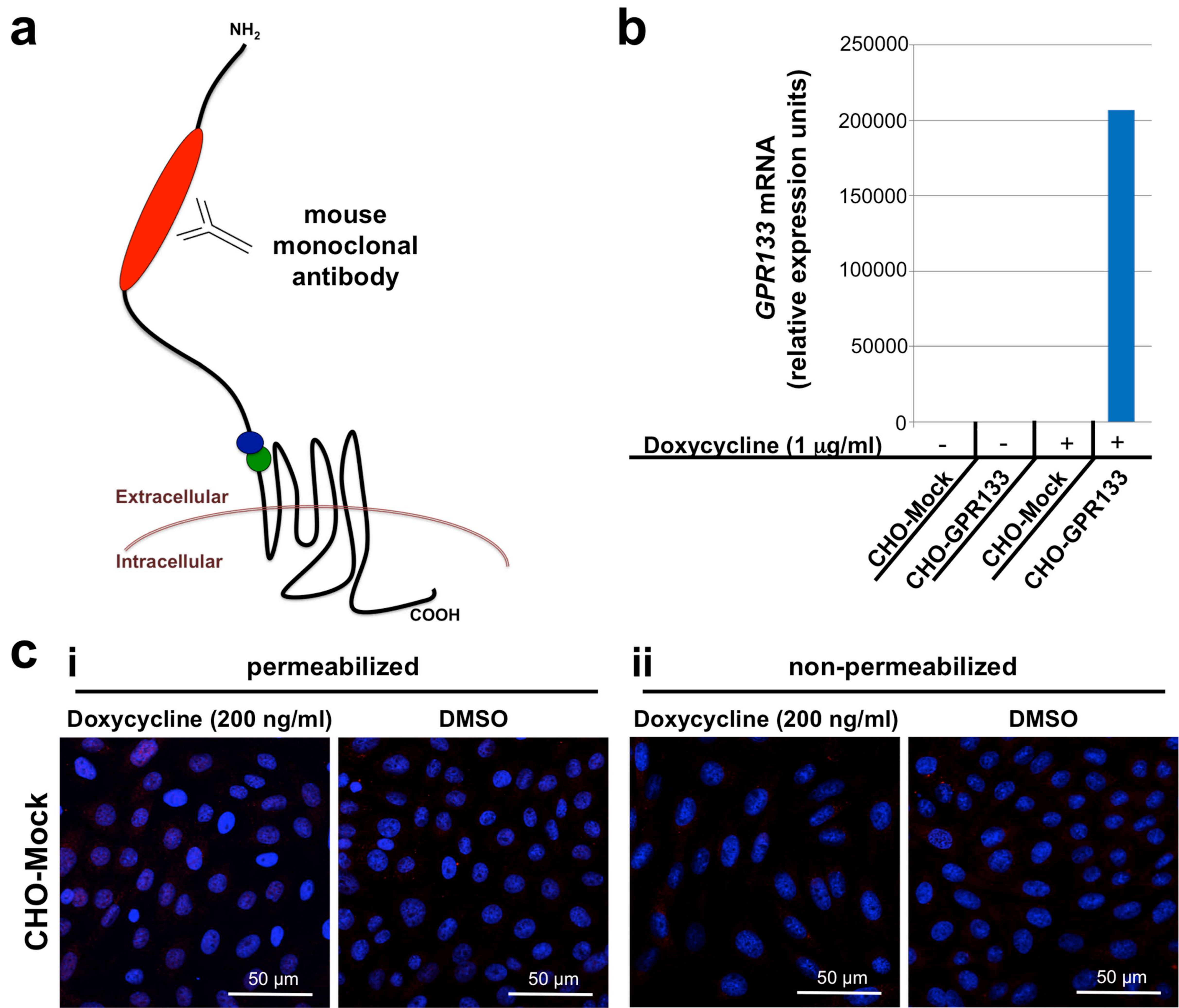

Supplementary Figure 5

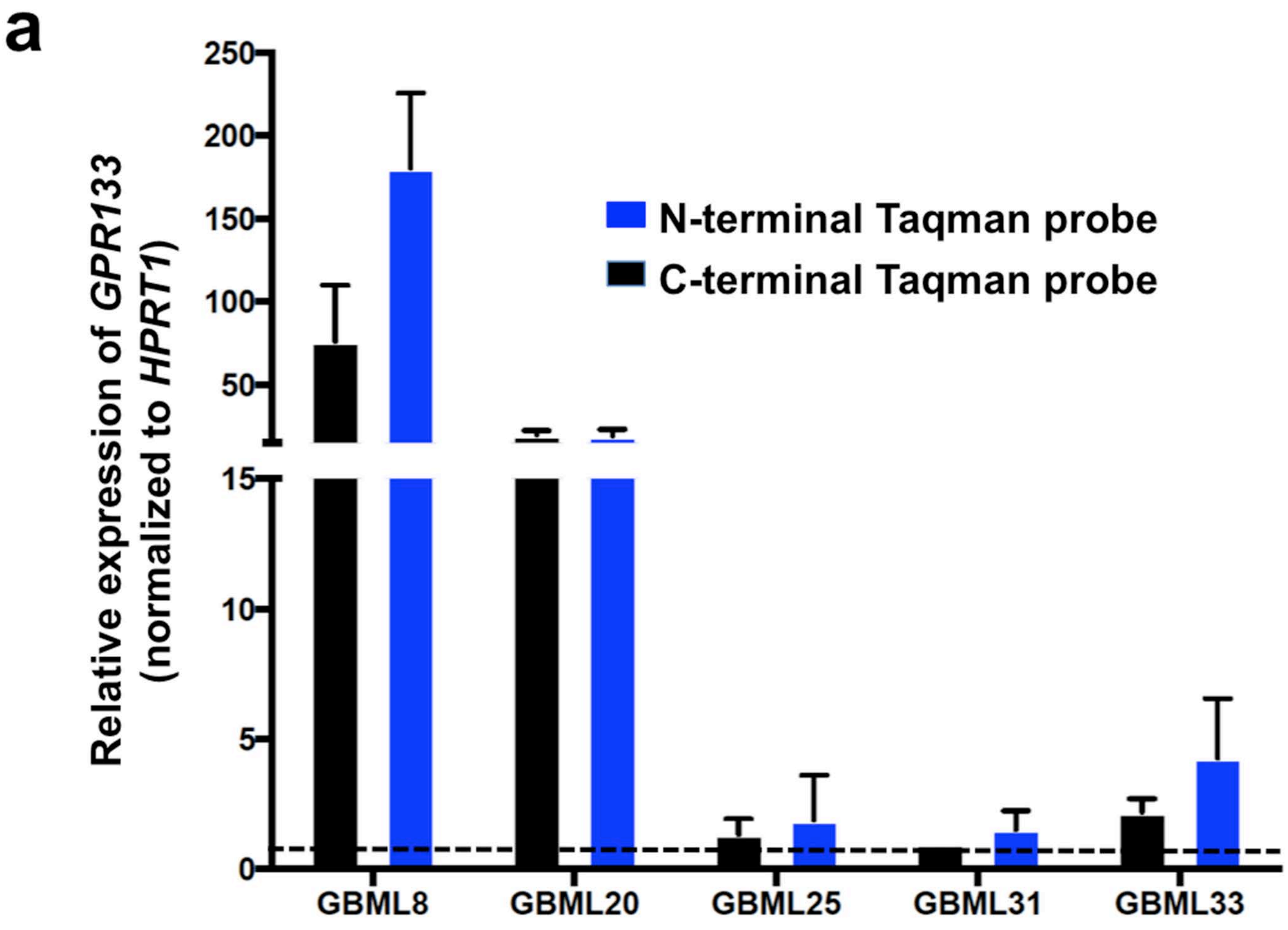

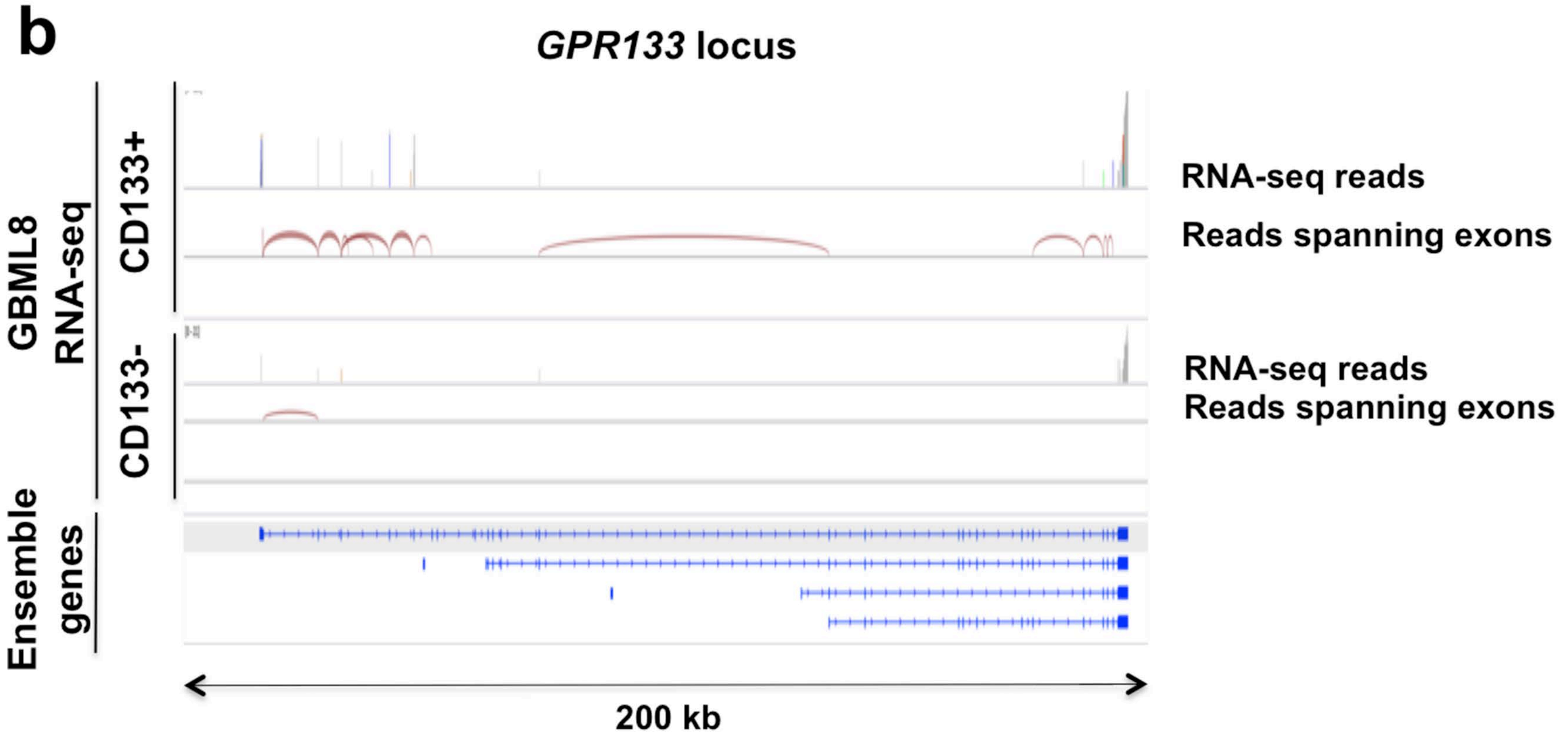

Supplementary Figure 6

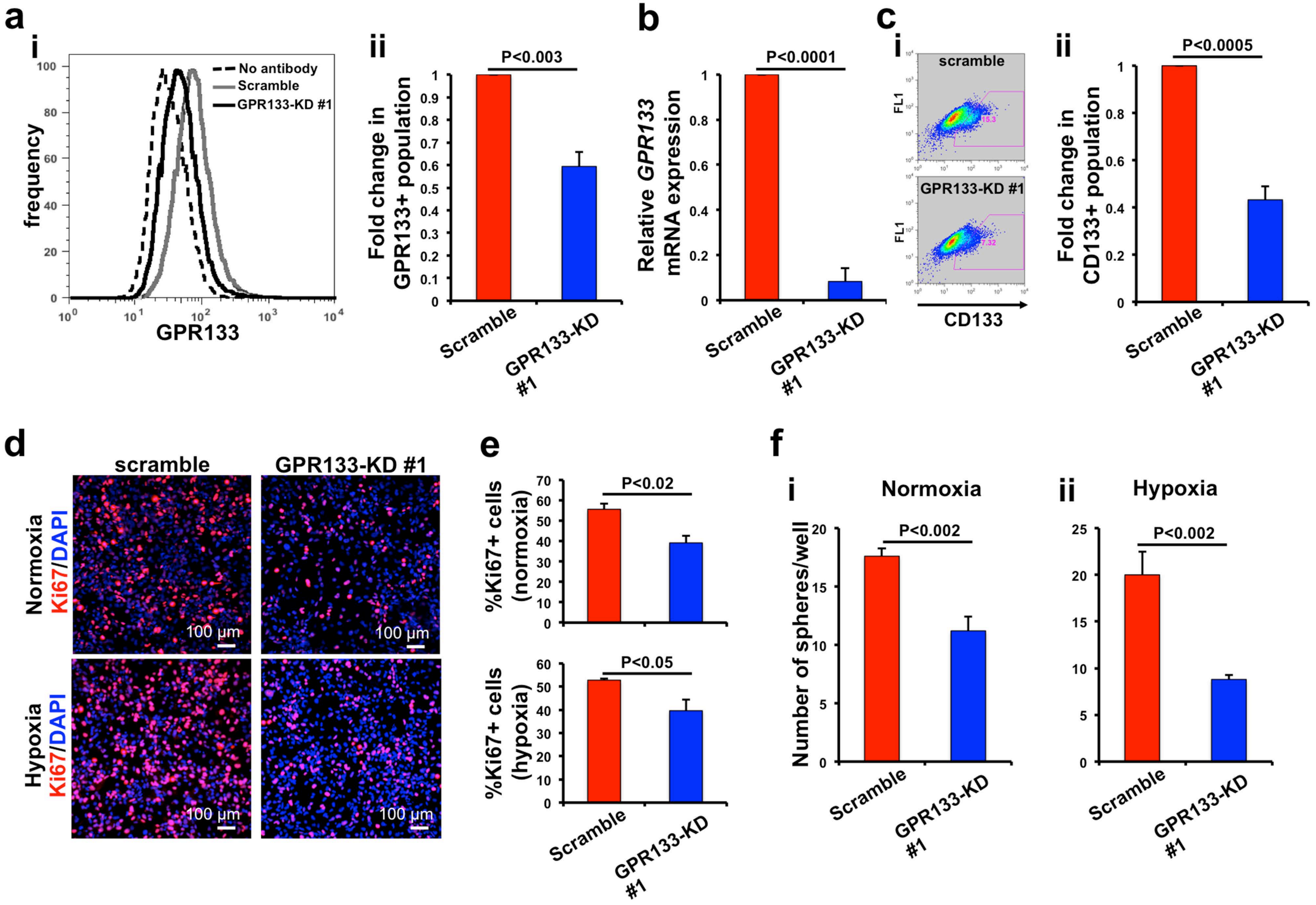

Supplementary Figure 7

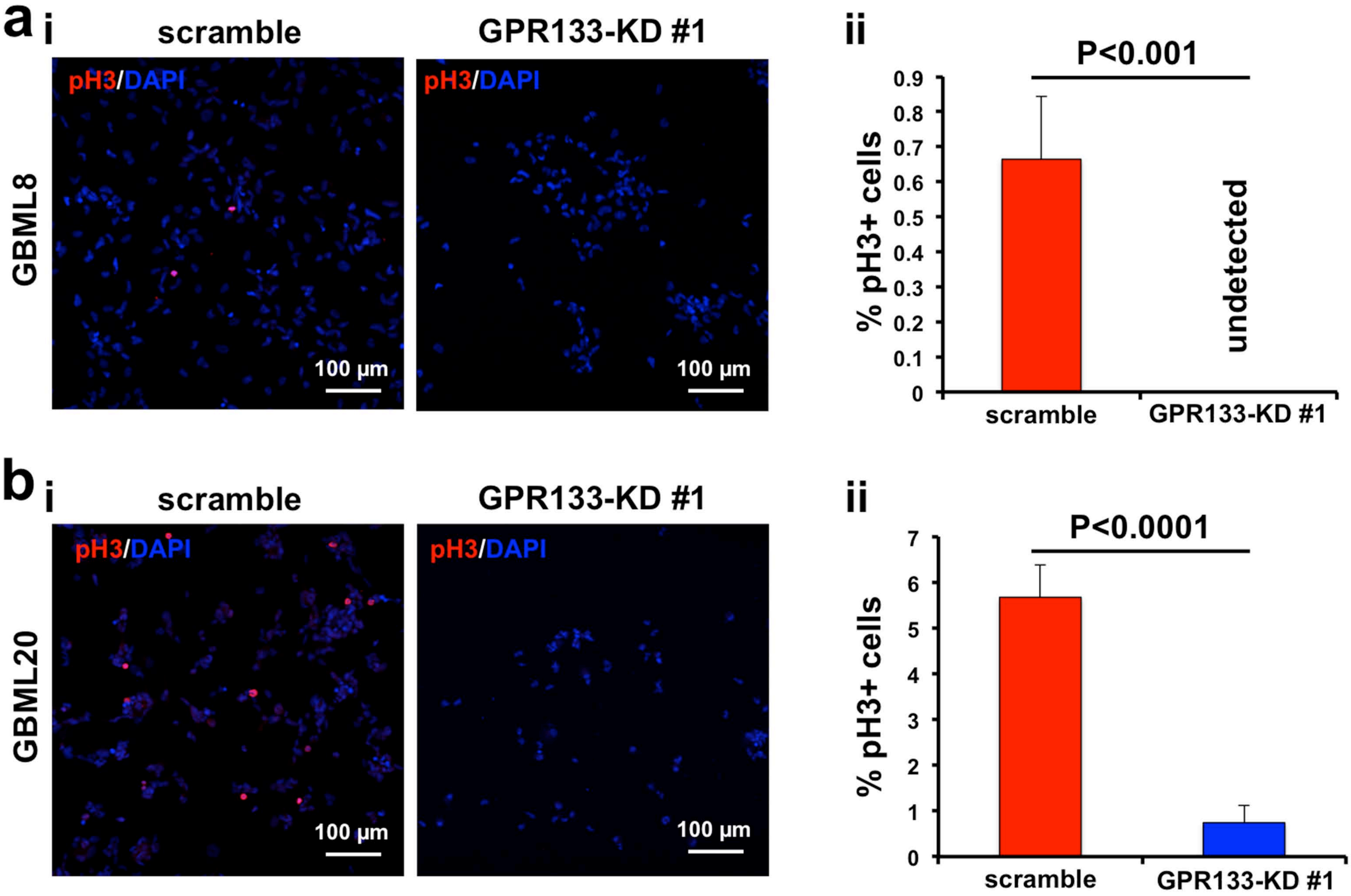

Supplementary Figure 8

a i

GBML8

Normoxia

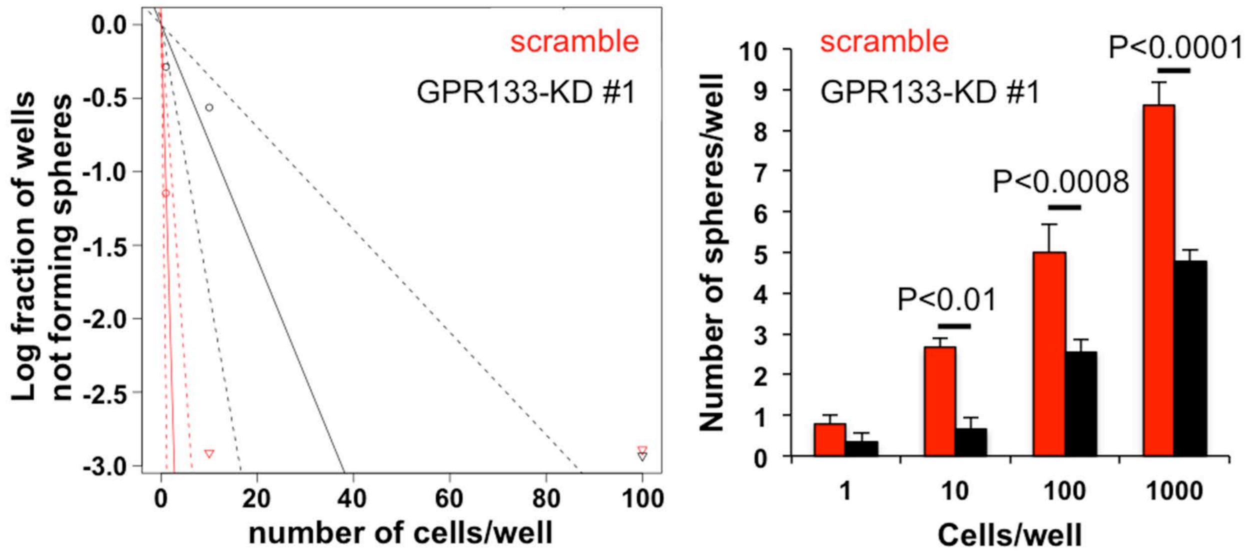

	Stem cell frequency	P-value
**Scramble**	1/1.5	P<0.00001
**GPR133-KD #1**	1/13	

ii

Hypoxia

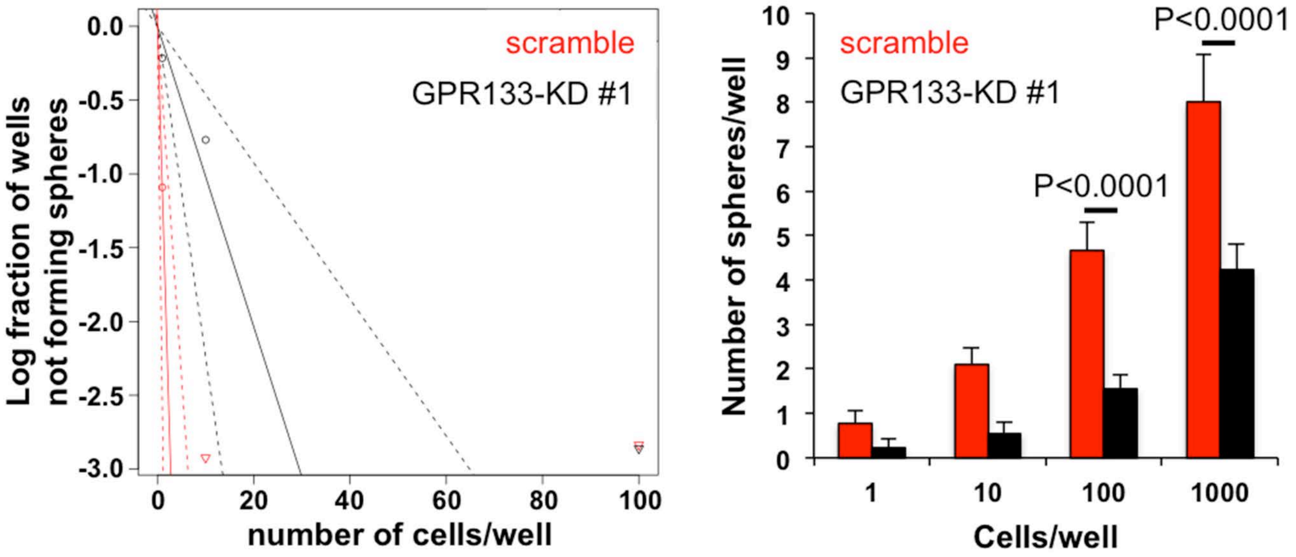

	Stem cell frequency	P-value
**Scramble**	1/1.5	P<0.00005
**GPR133-KD #1**	1/10.5	

b i

GBML20

Normoxia

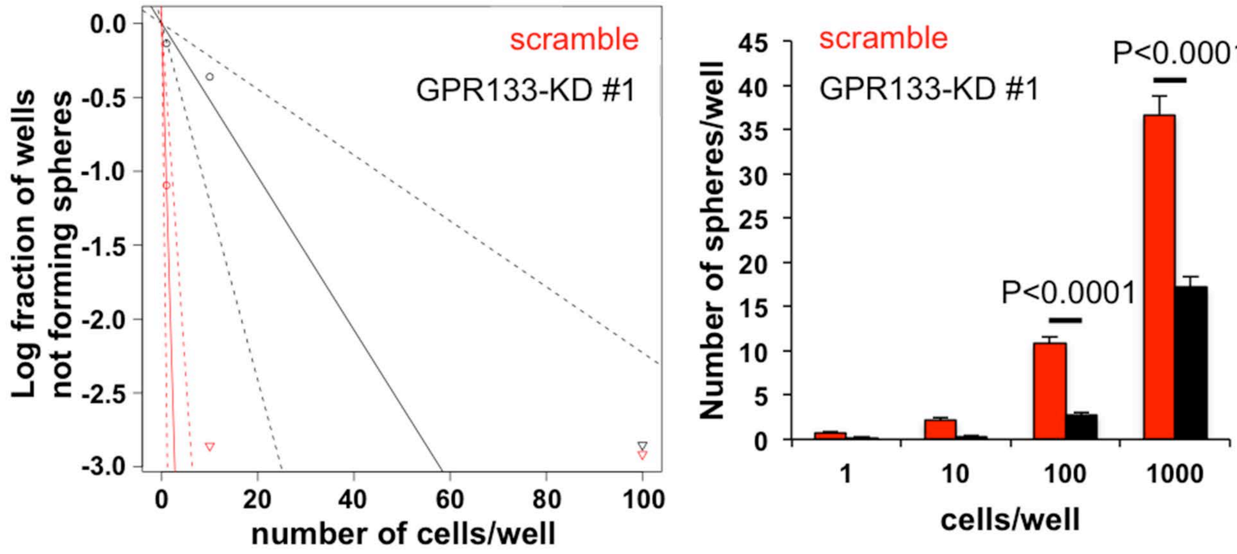

	Stem cell frequency	P-value
**Scramble**	1/1.5	P<0.0000006
**GPR133-KD #1**	1/19.8	

ii

Hypoxia

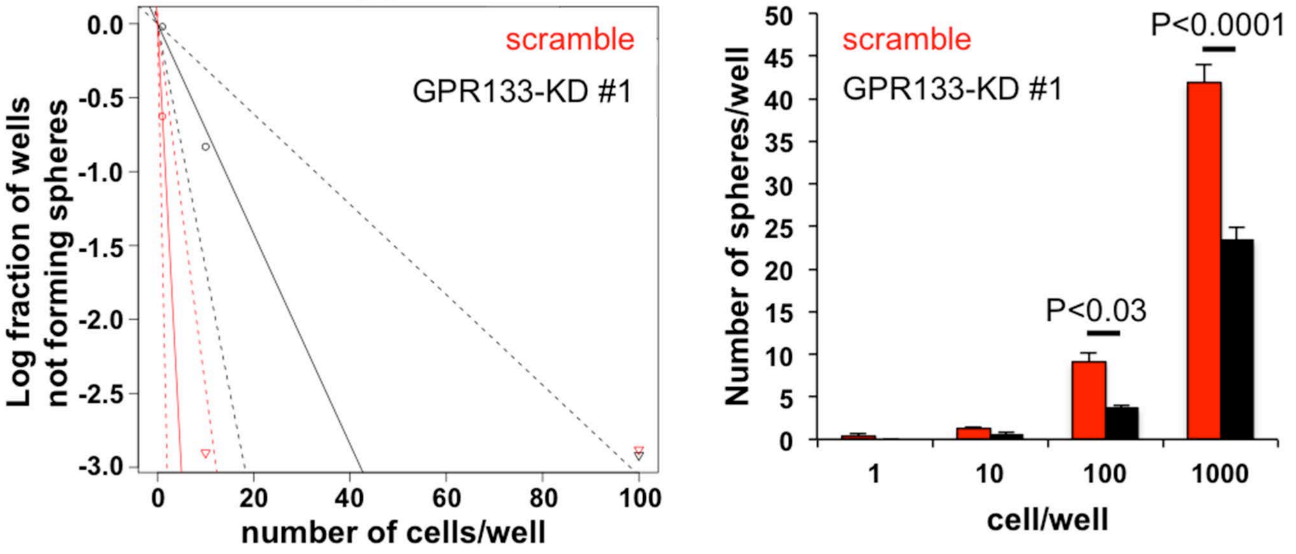

	Stem cell frequency	P-value
**Scramble**	1/1.5	P<0.000007
**GPR133-KD #1**	1/14.6	

c

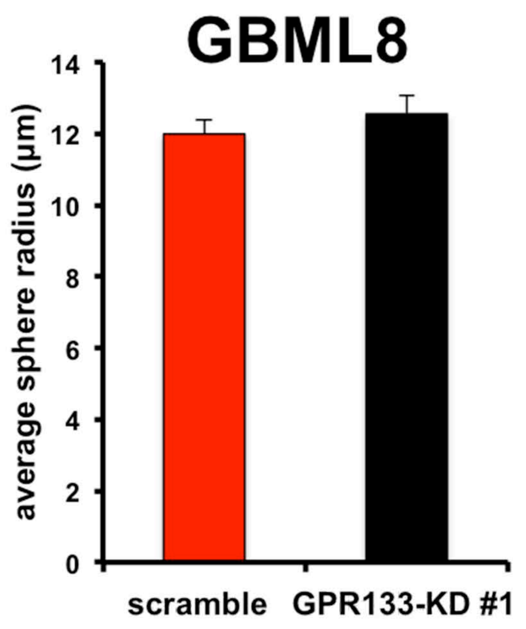

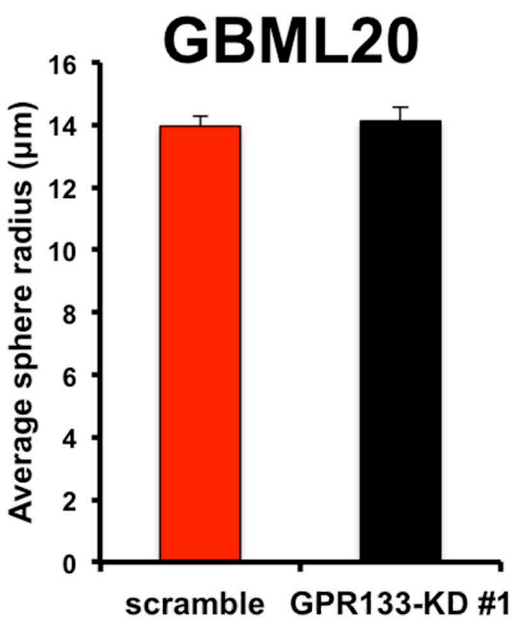

Supplementary Table 1a: Genes upregulated in CD133+ *vs.* CD133- GBM cells

gene	log2 change	fold change	FDR-adjusted p value
PROM1	4.02359	16.26377211	0.004
ACAN	3.77966	13.73380995	0.004
IGSF21	3.6893	12.90000751	0.004
ALPK2	3.50818	11.37803879	0.025
FAM180A	3.4572	10.98299791	0.004
SORCS3	3.37402	10.36767145	0.004
ANO1	3.17698	9.044119158	0.004
NPTX1	3.15352	8.898239933	0.004
ESPN	3.15049	8.879571141	0.016
CA9	3.07609	8.433257464	0.004
GPR133	3.0061	8.033897194	0.004
FAM150B	2.98885	7.938409582	0.004
SPNS2	2.94832	7.718497304	0.004
DHRS3	2.92511	7.595316059	0.047
SLC6A6	2.89976	7.463022316	0.004
NRXN3	2.82332	7.077893236	0.004
EDN2	2.67708	6.39560128	0.007
ADCY8	2.56274	5.908287377	0.004
RHBDF2	2.55968	5.895769001	0.004
PCDH8	2.46046	5.503921906	0.004
SHANK2	2.45849	5.49641143	0.004
GRIP2	2.30228	4.932366489	0.004
CHI3L1	2.25978	4.789184449	0.045
GMPR	2.24373	4.736199986	0.004
HAPLN3	2.21446	4.641078179	0.004
SORCS2	2.17459	4.514574438	0.004
LOC154761	2.11182	4.322362289	0.004
TPPP3	2.10823	4.3116199	0.004
SPAG4	2.10036	4.288163755	0.007
COL15A1	2.09563	4.274127662	0.004
LOXL2	2.08287	4.236491575	0.004
SLC14A1	2.08245	4.235258419	0.004
CAV1	2.05299	4.149650983	0.004
CDH5	2.05091	4.14367255	0.004
PRSS35	2.04238	4.119245183	0.036
MMP17	2.03999	4.112426801	0.004
HIC1	2.0392	4.110175509	0.023
MAP7D2	2.02911	4.081529826	0.004

LMO1	2.0216	4.060338483	0.004
SMAD7	2.00634	4.017616893	0.004
ESRRB	1.999	3.997228372	0.016
MT1E	1.99553	3.987625708	0.018
C1QTNF5	1.99517	3.986630788	0.007
OLFM1	1.98693	3.963925918	0.004
TNFAIP2	1.98364	3.954896665	0.048
EDN1	1.98353	3.954595131	0.011
LOC284578	1.98208	3.950622509	0.007
GABRG3	1.98182	3.949910599	0.018
NELL2	1.97029	3.918468773	0.004
FAM50B	1.95733	3.883426067	0.034
OLFML2A	1.9427	3.844244242	0.004
AQP1	1.93211	3.816129156	0.023
H1F0	1.92254	3.790898948	0.004
NDRG1	1.91194	3.763147922	0.004
APLN	1.8974	3.72541204	0.004
ELFN1	1.89274	3.713398127	0.013
WNT7A	1.86552	3.643992526	0.004
STC1	1.84502	3.592579295	0.004
NXPH4	1.84486	3.592180887	0.004
CDH7	1.84446	3.591185061	0.018
MYO1B	1.83421	3.565760981	0.004
OGDHL	1.81885	3.527998628	0.004
MAP3K5	1.80557	3.49567243	0.004
SNCB	1.78044	3.435309303	0.004
DUSP26	1.7681	3.406050912	0.039
SERPINF1	1.75522	3.375777926	0.007
OPCML	1.74628	3.354923802	0.004
PCSK1	1.7144	3.281601372	0.004
KCNK3	1.71425	3.281260195	0.018
SELM	1.71122	3.274376006	0.025
DDIT4L	1.69559	3.239093245	0.004
AMPD3	1.67555	3.194411122	0.004
INHBB	1.67535	3.193968313	0.004
CEBPD	1.67203	3.186626648	0.004
CDK18	1.66736	3.176328224	0.004
ADM	1.65296	3.144781975	0.004
WNT7B	1.64147	3.119835582	0.004
CYB5R2	1.63011	3.095365988	0.023
GALNTL4	1.625	3.084421651	0.004

ADAMTS7	1.6153	3.063753017	0.013
LGALS3	1.61346	3.059848026	0.004
SIK1	1.60679	3.045734107	0.004
FOSL2	1.60012	3.031685291	0.004
CAPG	1.5931	3.016969266	0.004
SGIP1	1.58736	3.004989604	0.004
NRP1	1.57982	2.989325506	0.004
ACCS	1.57952	2.988703958	0.021
MRVI1	1.5783	2.98617766	0.004
KBTBD11	1.57501	2.979375578	0.004
SLC16A3	1.573	2.975227525	0.004
CRABP2	1.5722	2.973578166	0.004
HSF4	1.5596	2.947721039	0.004
AKAP12	1.55173	2.931684799	0.004
COL23A1	1.54094	2.909840348	0.032
HSD11B2	1.54088	2.909719334	0.039
CNGA3	1.53308	2.894030246	0.013
BAMBI	1.52091	2.869720041	0.039
ALDOC	1.51809	2.86411615	0.004
VWA1	1.47682	2.783345494	0.011
EGLN3	1.47663	2.782978957	0.004
CHST15	1.47479	2.779431834	0.021
MFSD4	1.47137	2.772850818	0.007
PYGL	1.47036	2.770910283	0.004
PDZD2	1.46249	2.755835932	0.004
IL17RC	1.46152	2.753983661	0.045
ITPR1	1.44994	2.731966892	0.004
CLIC6	1.43228	2.69872879	0.038
RNF182	1.42411	2.683489048	0.004
GSN	1.41736	2.670963017	0.004
EFR3B	1.41381	2.664398731	0.004
ADSSL1	1.40184	2.642383745	0.004
INSIG2	1.39879	2.636803386	0.004
S1PR3	1.39323	2.62666097	0.004
TCEAL5	1.39179	2.624040524	0.007
CA12	1.38766	2.61653943	0.004
EPB49	1.36479	2.575388356	0.034
MLLT4-AS1	1.36347	2.573033071	0.021
FAM69C	1.36208	2.570555213	0.021
TF	1.34861	2.546666424	0.030
BHLHE40	1.34337	2.537433477	0.004

PPFIA4	1.34201	2.535042616	0.004
FAM110C	1.3388	2.52940841	0.047
BEST3	1.33022	2.514410148	0.004
MIAT	1.31799	2.493185107	0.004
NT5E	1.3026	2.466730322	0.004
FAM115C	1.29649	2.456305491	0.004
FGF11	1.2949	2.453599878	0.038
OBSL1	1.29456	2.453021706	0.004
ELL2	1.28918	2.4438911	0.004
TMEM123	1.2846	2.436144991	0.004
ZNF395	1.28442	2.435841061	0.004
PCOLCE2	1.28285	2.43319172	0.007
ID2	1.27942	2.427413691	0.004
SLC2A1	1.2714	2.413957038	0.004
STAC2	1.26583	2.404655129	0.004
LOC100216479	1.26444	2.40233942	0.004
GPR12	1.26042	2.395654735	0.004
PFKFB4	1.24961	2.377771366	0.004
HSPB6	1.24708	2.373605212	0.004
RCOR2	1.2457	2.371335842	0.004
MAF	1.24142	2.364311296	0.004
GATM	1.23682	2.35678475	0.004
ZNF334	1.23033	2.346206505	0.023
STC2	1.21213	2.316794367	0.004
NTRK2	1.20587	2.30676334	0.004
SLC43A3	1.20298	2.302147068	0.004
EHD2	1.19712	2.292815075	0.004
SH2D4A	1.19615	2.291274012	0.016
IGFBP5	1.19567	2.290511808	0.004
GYPC	1.19441	2.288512227	0.007
FAM49A	1.19233	2.285215151	0.004
MGST1	1.18968	2.281021429	0.045
HILPDA	1.18768	2.277861453	0.004
PTPRD	1.17923	2.2645588	0.004
SEMA5B	1.17591	2.259353479	0.016
SLC2A3	1.16547	2.24306278	0.004
LMO2	1.16336	2.239784608	0.018
NRN1	1.14803	2.216110778	0.004
YWHAH	1.13928	2.202710659	0.004
EMP2	1.13889	2.202115287	0.004
GABBR2	1.13096	2.19004422	0.004

ARHGEF3	1.12941	2.187692548	0.004
CNR1	1.12917	2.187328644	0.004
SFXN3	1.12198	2.176454711	0.007
KIAA1671	1.11606	2.167542077	0.004
TSC22D3	1.11344	2.163609294	0.004
MAFB	1.11026	2.158845501	0.034
EFS	1.10827	2.155869723	0.004
CD9	1.10732	2.154450572	0.004
PSTPIP2	1.09879	2.141749869	0.045
LOC254559	1.09859	2.14145298	0.030
PPP1R3C	1.0874	2.124907441	0.004
ST3GAL1	1.08427	2.120302344	0.004
PRPH	1.08074	2.115120709	0.043
FABP5	1.07707	2.109746997	0.004
PLOD2	1.06946	2.098647697	0.004
RARA	1.05355	2.075631017	0.030
TRIM2	1.04904	2.069152531	0.004
MED15	1.04897	2.069052138	0.004
CD97	1.04399	2.061922345	0.004
FKBP9L	1.03953	2.055557886	0.036
HK2	1.03389	2.047537678	0.004
PDK1	1.03177	2.044531088	0.004
NFATC4	1.02656	2.037160995	0.013
TET1	1.02306	2.032224802	0.011
SPEG	1.01486	2.020706793	0.028
MEST	1.00816	2.011344214	0.004
PPAP2B	1.00257	2.003565952	0.004
MT1X	1.00088	2.001220311	0.038
EPAS1	0.995335	1.993543381	0.004
ENO2	0.993995	1.991692603	0.004
TMEM45A	0.991327	1.988012737	0.007
B3GALNT1	0.98677	1.981743164	0.004
CHPF	0.983777	1.977636122	0.004
LINGO1	0.983445	1.977181071	0.004
CSPG4	0.98313	1.976749418	0.011
GRIK1	0.978111	1.969884442	0.004
TGFA	0.976867	1.968186591	0.004
SIPA1L2	0.975836	1.966780559	0.004
FLNB	0.968271	1.956494426	0.018
CD109	0.964911	1.951943102	0.023
LRRC49	0.961732	1.947646702	0.007

MLLT3	0.955775	1.939621297	0.034
VEGFA	0.955466	1.939205909	0.004
MXI1	0.938912	1.917081937	0.004
MIR210HG	0.935642	1.912741617	0.039
ZFP36L2	0.932293	1.908306624	0.004
SYTL2	0.930037	1.90532486	0.004
LGR6	0.926289	1.900381411	0.034
TMEM198	0.925968	1.899958623	0.043
MDK	0.9257	1.899605713	0.004
APOLD1	0.918636	1.890327233	0.004
FLJ46906	0.9126	1.882434935	0.007
RND3	0.912391	1.882162251	0.045
KCNE4	0.911253	1.880678184	0.004
SORL1	0.904571	1.871987758	0.004
SCARB1	0.902987	1.869933546	0.011
NPNT	0.902121	1.868811426	0.013
FKBP9	0.899069	1.864862162	0.004
NT5C3	0.896159	1.861104417	0.004
PDE4D	0.894443	1.85889206	0.032
C1orf198	0.885987	1.848028492	0.004
PPARGC1B	0.885446	1.847335625	0.004
FGFR1	0.869414	1.826920683	0.004
CABIN1	0.863656	1.819643716	0.013
ERO1L	0.858669	1.813364567	0.018
MEG3	0.858232	1.812815372	0.023
IFITM3	0.857451	1.811834274	0.028
LOC730101	0.855987	1.809996616	0.043
FRAS1	0.848884	1.801107136	0.004
AMD1	0.846678	1.798355199	0.004
TMEM22	0.843261	1.794100863	0.025
RORA	0.843026	1.793808647	0.013
MT3	0.842154	1.792724753	0.004
SBF2	0.841085	1.791396882	0.004
ARL4C	0.84046	1.790620986	0.004
IQSEC1	0.839238	1.789104926	0.032
HJURP	0.836282	1.785442905	0.013
C11orf41	0.829182	1.77667771	0.023
EYA2	0.828153	1.77541095	0.025
NOTCH3	0.826995	1.773986462	0.013
KATNAL1	0.826141	1.772936666	0.018
CSGALNACT1	0.819128	1.764339261	0.011

PSMD5	0.814537	1.758733636	0.021
S1PR2	0.814434	1.758608077	0.050
JAM2	0.8112	1.75467033	0.032
RP9P	0.80781	1.7505521	0.030
CXCR4	0.806631	1.749122097	0.025
VOPP1	0.796003	1.736284066	0.007
NCALD	0.794744	1.73476952	0.011
CAV2	0.78674	1.725171754	0.021
C14orf43	0.785832	1.724086311	0.016
NEDD4L	0.783422	1.721208655	0.023
GRB10	0.782665	1.720305752	0.007
TNIP1	0.773008	1.708828958	0.023
TSKU	0.771003	1.706455745	0.043
AK4	0.758256	1.691444688	0.004
PPFIBP2	0.754097	1.686575614	0.023
LPL	0.75126	1.683262292	0.016
CCNG2	0.74887	1.680476071	0.045
FEM1C	0.737755	1.667578874	0.039
DDIT4	0.711344	1.637328726	0.036
PSPH	0.702172	1.626952352	0.016
C5orf62	0.687785	1.610808511	0.038
BNIP3L	0.662611	1.582944858	0.043
ARHGAP21	0.660533	1.580666489	0.045

Supplementary Table 1b: Genes downregulated in CD133+ *vs.* CD133- GBM cells

gene	log2 change	fold change	FDR-adjusted p value
KCNJ6	2.82636	7.092823266	0.004
LRRN1	2.40211	5.285756626	0.004
ID3	2.35582	5.118850954	0.004
IL33	2.2916	4.895987925	0.011
SCRG1	1.97385	3.928149942	0.004
NEUROD1	1.86324	3.638238197	0.004
ID1	1.75405	3.37304134	0.004
ST6GAL1	1.67493	3.193038614	0.004
SPP1	1.5431	2.914200218	0.004
SNCAIP	1.52535	2.87856542	0.011
GRIK3	1.47713	2.783943631	0.004
GPR126	1.39706	2.633643372	0.039
EDNRB	1.39034	2.621404521	0.004
DCC	1.38744	2.616140458	0.016
HOPX	1.36608	2.577692195	0.013
TMEM108	1.35151	2.551790695	0.004
IQGAP2	1.29641	2.456169288	0.004
TIMP3	1.26842	2.408975966	0.032
KIAA1598	1.22756	2.341706069	0.004
BCAN	1.22132	2.331599504	0.004
PLAT	1.20322	2.302530074	0.004
CDH6	1.19191	2.284549972	0.004
POSTN	1.17074	2.251271415	0.004
VGF	1.16919	2.248853998	0.004
TNFRSF19	1.14168	2.206378035	0.004
HSPA12A	1.07027	2.099826312	0.004
SLC1A2	1.05194	2.073315973	0.007
KCNQ2	1.03411	2.047849936	0.004
CD24	1.01029	2.014315962	0.004
LAMB1	0.957362	1.941756102	0.018
FLRT3	0.944393	1.924379052	0.013
PVRL3	0.937611	1.915353922	0.004
BDNF	0.93551	1.912566618	0.041
VCAN	0.903931	1.871157501	0.048
SHROOM3	0.887019	1.849350912	0.013
LRRC17	0.885076	1.84686191	0.041
FST	0.884673	1.846346083	0.048
RCAN1	0.873007	1.831476259	0.016

ANXA1	0.871862	1.830023278	0.004
ODZ2	0.869303	1.826780127	0.016
C8orf46	0.800004	1.741105954	0.048
ALCAM	0.77189	1.707505233	0.034
WISP1	0.754421	1.686954427	0.032
EYA4	0.741246	1.671618928	0.023
SACS	0.740583	1.670850901	0.021
DLC1	0.725233	1.653167607	0.038
COL5A2	0.719459	1.646564469	0.039
SPRY4	0.708049	1.633593465	0.03

Supplementary Table 2a. Taqman Assays

Target	TaqMan Assay
GPR133-C-terminal	Hs00914797_m1
GPR133-N-terminal	Hs00138665_m1
HPRT1	Hs28000695_m1
GAPDH	Hs02758991_m1
PROM1 (CD133)	Hs00195682_m1
HIF1A	Hs00153153_m1
18S	Hs99999901_s1

Supplementary Table 2b. PCR primers used in the study

Target	Forward primer	Reverse primer	Amplicon (bp)
GPR133 (in CHO cells)	5'CCAGAGACAACAGCATGACC3'	5'TTTGGACTTCCAGGTGAACA3'	86
GPR133 promoter HRE	5'CTGTGTGTCCACGTGTATTTGG3'	5'AGAAAAGAGTCAGCCCCTTCG3'	90
CA9 promoter HRE	5'GACTTTGGCTCCATCTCTGC3'	5' GTGTGTACGGGCTGTACGTG3'	133

Supplementary Table 2c. shRNA constructs

shRNA construct	Target sequence
GPR133 target #1	5'CCTGCAGGGACTGTTCATATT3'
GPR133 target #2	5'GGAGTCACGCTTCTCTATTAC3'
HIF1A	5'GCCGCTCAATTTATGAATATT3'
scramble	5'CCTAAGGTTAAGTCGCCCTCG3'

Supplementary Table 3. Antibodies used for Immunofluorescence staining

Antigen	Company/Catalog #	Dilution
**GPR133 (polyclonal)**	LSBio/ LS-A2034	1:200
**GPR133 (monoclonal)**		1:1000 (1:5000 in CHO cell assays)
**CD133 (W6B3C1)**	Miltenyi/130-092-395	1:50
**Ki67**	Abcam/ ab15580	1:200
**pH3**	EMD Millipore/06-570	1:1000
**Hif1α**	Bethyl Labs/ A300-286A	1:200
**CA9**	Abcam/ ab15086	1:200
**Hydroxy-probe (pimonidazole)**	HydroxyProbe/ HP1-1000	1:100
**hNA**	Millipore/ MAB1281	1:200
